# Supplementary material for: Assessing the safety of home oximetry for COVID-19: a multisite retrospective observational study
Source: BMJ Open. 2021 Sep 14;11(9):e049235. doi: 10.1136/bmjopen-2021-049235 (PMC8441226; doi:10.1136/bmjopen-2021-049235)
Supplement: Supplementary data [file bmjopen-2021-049235supp001.pdf]

Category	Variable	Source	Data Type
Baseline Data	Age	PDS	Numeric
	Ethnicity	PDS	Categorical
	Sex	PDS	Categorical
	post code	PDS	String
	occupation	Onboarding	Categorical /string
	Registered GP Practice	PDS	String
	Home circumstances	Onboarding	Categorical
	Smoker	Onboarding / GP	Categorical
	**Clinical Comorbidities:***		
	BMI	Onboarding / GP	Numeric
	Asthma	Onboarding / GP	Binary
	COPD	Onboarding / GP	Binary
	Emphysema	Onboarding / GP	Binary
	Bronchiectasis	Onboarding / GP	Binary
	Other Chronic Resp Disease	Onboarding / GP	Binary / string
	Chronic cardiac disease	Onboarding / GP	Binary
	Chronic liver disease	Onboarding / GP	Binary
	Stroke or dementia	Onboarding / GP	Binary
	Neurological	Onboarding / GP	Binary
	Chronic kidney disease	Onboarding / GP	Binary
	Hypertension	Onboarding / GP	Binary
	Diabetes	Onboarding / GP	Binary
	Cancer	Onboarding / GP	Binary
	Cancer site(s) / types	Onboarding / GP	String
	Date of Cancer Diagnosis	Onboarding / GP	Date
	HIV	Onboarding / GP	Binary
	Transplant receipient	Onboarding / GP	Binary
	Transplant sites	Onboarding / GP	Cateogrical
	Immunosuppression regimen	Onboarding / GP	String
	**Onset of Symptoms:**		

Initial Entry	Breathlessness	Onboarding	Date
	Myalgia	Onboarding	Date
	Chills	Onboarding	Date
	Severe fatigue	Onboarding	Date
	Sputum	Onboarding	Date
	Dizziness	Onboarding	Date
	Cough	Onboarding	Date
	Nausea or vomiting	Onboarding	Date
	Diarrhoea	Onboarding	Date
	Headache	Onboarding	Date
	Sore throat	Onboarding	Date
	Nasal congestion	Onboarding	Date
	Anosmia	Onboarding	Date
	Unwell contact	Onboarding	Binary
	Sars COV 2 swab positive	Onboarding	Categorical
	Referral source	Onboarding	Categorical
	Date and time of recording	Onboarding	Timestamp
	**Clinical observations:**		
	O2 sats at rest	Onboarding	Numerical
	O2 sats after exertion	Onboarding	Numerical
	Heart rate	Onboarding	Numerical
	Respiratory rate	Onboarding	Numerical
	Blood pressure	Onboarding	Numerical
	Conscious level (GCS)	Onboarding	Numerical
	Temperature	Onboarding	Numerical
	NEWS Score	Onboarding	Numerical

Diary Entry (iterates 2-3x daily)	Date and time of recording	Diary	Timestamp
	**Clinical observations:**		
	O2 sats at rest	Diary	Numerical

	O2 sats after exertion	Diary	Numerical
	HR	Diary	Numerical
	Temperature	Diary	Numerical

Diary Aggregate Data (N.B. Derived from Diary entries - not collected in itself)	*Mean pulse*	*Calculated from Diary*	*Numeric*
	Mean O2 sats	*Calculated from Diary*	*Numeric*
	Datetime of lowest O2 Sats	*Calculated from Diary*	*Numeric*
	Value of lowest O2 Sats	*Calculated from Diary*	*Numeric*
	Datetime of highest HR	*Calculated from Diary*	*Numeric*
	Value of highest HR	*Calculated from Diary*	*Numeric*
	Datetime of highest Temp	*Calculated from Diary*	*Numeric*
	Value of highest Temp	*Calculated from Diary*	*Numeric*
	Number of days of fever	*Calculated from Diary*	*Numeric*
	Days on Virtual Ward	*Calculated from Diary*	*Numeric*

In Hospital Data (one for each A&E presentation or hospital admission)	First location on arrival	Hospital EHR / HES	Categorical
	Date and time of arrival	Hospital EHR / HES	Timestamp
	Admitted from A&E	Hospital EHR / HES	Binary
	**Obs on arrival:**		
	O2 sats on air	Hospital EHR	Numeric
	O2 desturation (>3%)	Hospital EHR	Numeric
	Heart rate	Hospital EHR	Numeric
	Respiratory rate	Hospital EHR	Numeric
	Blood pressure	Hospital EHR	Numeric
	Conscious level (GCS)	Hospital EHR	Numeric
	Temperature	Hospital EHR	Numeric
	NEWS Score	Hospital EHR	Numeric
	**Treatment given:**		

	Highest Oxygen Requirement	Hospital EHR	Numeric
	High flow oxygen	Hospital EHR	Binary
	CPAP	Hospital EHR	Binary
	NIV	Hospital EHR	Binary
	Intubation	Hospital EHR	Binary
	level 2 care	Hospital EHR	Binary
	Level 3 care (ITU)	Hospital EHR	Binary
	**Outcomes:**		
	Date and time of discharge or in-hospital d	Hospital EHR / HES	Timestamp
	Discharge destination	Hospital EHR / HES	Categorical
	Length of Stay	*Computed from in-hospital data*	*Numeric*

Mortality	Date of Death	ONS	Date
	Cause(s) of Death	ONS	String
