# Supplementary material for: Assessing the safety of home oximetry for COVID-19: a multisite retrospective observational study
Source: BMJ Open. 2021 Sep 14;11(9):e049235. doi: 10.1136/bmjopen-2021-049235 (PMC8441226; doi:10.1136/bmjopen-2021-049235)
Supplement: Author's manuscript [file bmjopen-2021-049235.draft_revisions.pdf]

BMJ Open

BMJ Open is committed to open peer review. As part of this commitment we make the peer review history of every article we publish publicly available.

When an article is published we post the peer reviewers' comments and the authors' responses online. We also post the versions of the paper that were used during peer review. These are the versions that the peer review comments apply to.

The versions of the paper that follow are the versions that were submitted during the peer review process. They are not the versions of record or the final published versions. They should not be cited or distributed as the published version of this manuscript.

BMJ Open is an open access journal and the full, final, typeset and author-corrected version of record of the manuscript is available on our site with no access controls, subscription charges or pay-per-view fees (http://bmjopen.bmj.com).

If you have any questions on BMJ Open's open peer review process please email info.bmjopen@bmj.com

BMJ Open

Assessing the Safety of Home Oximetry for Covid-19: A multi-site retrospective observational study.

Journal:	*BMJ Open*
Manuscript ID	bmjopen-2021-049235
Article Type:	Original research
Date Submitted by the Author:	19-Jan-2021
Complete List of Authors:	Clarke, Jonathan; Imperial College London, Centre for Mathematics of Precision Healthcare<br>Flott, Kelsey; Imperial College London, Institute of Global Health Innovation<br>Fernandez Crespo, Roberto; Imperial College London, Institute of Global Health Innovation<br>Ashrafian, Hutan; Imperial College London, Institute of Global Health Innovation<br>Fontana, Gianluca; Imperial College London, Institute of Global Health Innovation<br>Benger, Jonathan; NHS Digital<br>Darzi, Ara; Imperial College London, Institute of Global Health Innovation<br>Elkin, Sarah; Imperial College London, National Heart and Lung Institute
Keywords:	COVID-19, Telemedicine < BIOTECHNOLOGY & BIOINFORMATICS, PRIMARY CARE, PUBLIC HEALTH, RESPIRATORY MEDICINE (see Thoracic Medicine)

SCHOLARONE™
Manuscripts

I, the Submitting Author has the right to grant and does grant on behalf of all authors of the Work (as defined in the below author licence), an exclusive licence and/or a non-exclusive licence for contributions from authors who are: i) UK Crown employees; ii) where BMJ has agreed a CC-BY licence shall apply, and/or iii) in accordance with the terms applicable for US Federal Government officers or employees acting as part of their official duties; on a worldwide, perpetual, irrevocable, royalty-free basis to BMJ Publishing Group Ltd ("BMJ") its licensees and where the relevant Journal is co-owned by BMJ to the co-owners of the Journal, to publish the Work in this journal and any other BMJ products and to exploit all rights, as set out in our licence.

The Submitting Author accepts and understands that any supply made under these terms is made by BMJ to the Submitting Author unless you are acting as an employee on behalf of your employer or a postgraduate student of an affiliated institution which is paying any applicable article publishing charge ("APC") for Open Access articles. Where the Submitting Author wishes to make the Work available on an Open Access basis (and intends to pay the relevant APC), the terms of reuse of such Open Access shall be governed by a Creative Commons licence – details of these licences and which Creative Commons licence will apply to this Work are set out in our licence referred to above.

Other than as permitted in any relevant BMJ Author's Self Archiving Policies, I confirm this Work has not been accepted for publication elsewhere, is not being considered for publication elsewhere and does not duplicate material already published. I confirm all authors consent to publication of this Work and authorise the granting of this licence.

For peer review only

Assessing the Safety of Home Oximetry for Covid-19: A multi-site retrospective observational study.

Authors:

Jonathan Clarke[1]

Kelsey Flott[2]

Roberto Crespo[2]

Hutan Ashrafian[2]

Gianluca Fontana[2]

Jonathan Benger[3]

Ara Darzi[2]

Sarah Elkin[4]

Institution:

1. Centre for Mathematics of Precision Healthcare, Imperial College London, London, SW7 2AZ
2. Institute of Global Health Innovation, Imperial College London, London, SW7 2NA
3. NHS Digital, 1 Trevelyan Square, Boar Lane, Leeds, West Yorkshire. LS1 6AE.
4. National Heart and Lung Institute, Imperial College London, Guy Scadding Building, Cale Street, London, SW3 6LY

Word count:

3163

Corresponding Author

Dr Jonathan Clarke

Centre for Mathematics of Precision Healthcare, Imperial College London, London SW7 2AZ

j.clarke@imperial.ac.uk

+44 (0) 7792 690037

For peer review only

Abstract

Objectives

To determine the safety and effectiveness of home oximetry monitoring pathways safe for Covid-19 patients in the English NHS

Design

This was a retrospective, multi-site, observational study of home oximetry monitoring for patients with suspected or proven Covid-19 Setting This study analysed patient data from four Covid-19 home oximetry pilot sites in England across primary and secondary care settings.

Participants

1338 participants were enrolled in a home oximetry programme at one of the four pilot sites. Participants were excluded if primary care data and oxygen saturations are rest at enrolment were not available. 908 participants were included in the analysis.

Interventions

Home oximetry monitoring was provided to participants with a known or suspected diagnosis of Covid-19. Participants were enrolled following attendance to accident and emergency departments, hospital admission or referral through primary care services.

Results

Of 908 patients enrolled into four different Covid-19 home oximetry programmes in England, 771 (84.9%) had oxygen saturations at rest of 95% or more, and 320 (35.2%) were under 65 years of age and without comorbidities. 52 (5.7%) presented to hospital and 28 (3.1%) died following enrolment, of which 14 (50%) had Covid-19 as a named cause of death. All-cause mortality was significantly higher in patients enrolled after admission to hospital (OR 8.70), compared to those enrolled in primary care. Patients enrolled after hospital discharge (OR 0.31) or emergency department presentation (OR 0.42) were significantly less likely to present to hospital than those enrolled in primary care.

Conclusions

This study find that home oximetry monitoring can be a safe pathway for Covid-19 patients; and indicates increases in risk to vulnerable groups and patients with oxygen saturations < 95% at enrolment, and in those enrolled on discharge from hospital. Findings from this evaluation have contributed to the national implementation of home oximetry across England.

For peer review only - http://bmjopen.bmj.com/site/about/guidelines.xhtml

Strengths and limitations of this study

- Several clinical pathways to remotely monitor patients with Covid-19 using home oximetry have been developed in England.
- This multisite study examines outcomes and variation between four of these new clinical pathways for the management of Covid-19 in England.
- It provides insight into the clinical status of patients enrolled under different home oximetry pathways and examines their rates of hospital attendance and all-cause mortality.
- The study is limited to four sites, and is unable to comment on the outcomes of patients not enrolled into these pathways or on the performance of pathways not included in the study.

For peer review only

Introduction

Before the outbreak of the Covid-19 pandemic, compelling evidence already existed regarding the role of telemedicine and digital technologies in restructuring how healthcare is delivered, indicating an opportunity to expand the use of virtual pathways (1). Such evidence suggests that care delivered remotely can, in many circumstances, safely meet patients' clinical needs and personal preferences (1)(2)(3). Specifically, remote monitoring pathways, those that rely on an initial point of contact with the health services followed by continuous monitoring via phone calls, digital or app-based diaries or wearable sensors, have also demonstrated effectiveness, especially when supported by behavioural change models (4)(5)(6). However, the clinical effectiveness, safety and economic utility of this type of monitoring is context-dependent and varies considerably across clinical conditions; therefore, more evidence is required to fully assess their impact (4).

During the Covid-19 pandemic, the UK National Health Service (NHS), and health systems across the world, rapidly adopted novel remote monitoring pathways, many relying on home pulse oximetry (7)(8)(9). These pathways were often initiated quickly in an attempt to proactively manage patients without risking unnecessary patient travel to hospital, but subsequently varied in terms of the devices used, patients included and the method of implementation. The available literature suggests potential for home management of Covid to support a positive patient experience (10)(11)(12). However, while pulse oximetry and trends over time appear to be an effective way of detecting deterioration, the evidence surrounding the safety of oximetry devices, specifically low-cost pocket oximeters, is variable and more research is required to understand what oxygen saturation thresholds should trigger a patient to seek in-person care (13)(14). Overall, the literature surrounding whether home oximetry monitoring for Covid-19 patients is safe remains inconclusive (9)(15).

In the UK, NHS England/Improvement, the body responsible for improved delivery of NHS care, in partnership with NHS Digital and Imperial College London, set out to understand, quantitatively, whether home oximetry monitoring was a safe clinical pathway to implement nationally. Following the first UK peak of Covid-19, in Spring 2020, pilots of home oximetry monitoring tested a system-wide approach to the early detection of Covid-19 in the community. As part of this pilot, a rapid evaluation was conducted to determine whether home oximetry monitoring was a safe clinical pathway. Based on existing literature, the initial hypothesis was that early recognition, escalation, admission and treatment could save lives in Covid-19 and would be a safe approach to adopt.

Aims & Objectives

This study aimed to answer the research question: *Are home oximetry monitoring pathways safe and effective for Covid-19 patients in the English NHS?*

The primary objective was to determine whether patients suffered any adverse consequences as a result of home oximetry monitoring. The secondary objective was to explore whether the recommendations relating to oxygen saturation that were published in June 2020 in the NHS Covid-19 assessment pathway were correct. The final objective was to contribute to a recommendation to NHS England/Improvement regarding the safety and suitability of home oximetry monitoring as a national Covid-19 pathway.

For peer review only - http://bmjopen.bmj.com/site/about/guidelines.xhtml

Methods

Setting & Design

To answer this question, Imperial College NHS Healthcare Trust, NIHR Imperial Patient Safety Translational Research Centre (PSTRC) at the Institute of Global Health Innovation (IGHI) at Imperial College London, working in partnership with NHS Digital, assessed existing evidence concerning home oximetry monitoring for respiratory conditions and analysed prospective data collected from four sites.

This was a retrospective, multi-site, observational study of home oximetry monitoring for patients with suspected or proven Covid-19 in England during Summer 2020, including an analysis of patient data from four pilot sites: North West London, Slough, South Tees and Watford.

Quantitative analysis was conducted in the IGHI's Big Data and Analytical Unit (BDAU). The BDAU provide a fully certified ISO 27001:2013 research environment within Imperial College and is fully compliant with NHS IG Toolkit Level 3 (EE133887). The work was conducted as a service evaluation, as institutional Research Governance deemed that it did not require further ethics committee approval. Information Governance approval was obtained and a data sharing agreement established (DARS-NIC-396113-N9L4L-v1.2). Analysis took place within the Imperial College London Big Data and Analytical Unit.

Information & Data

Four separate datasets linked by a pseudonymised patient identifier were provided by NHS Digital for the express purpose of this evaluation. A full list of variables collected is included in Appendix A and summarised below:

Primary Care Records: Data pertaining to patient demographics and clinical comorbidities were obtained from the General Practice Extraction Service (GPES) Data for Pandemic Planning and Research (GDPPR) for all patients enrolled into a home oximetry monitoring programme at one of the four sites. Records were available from the start of a patient's primary care record in the practice to the date of data transfer (25th Sept 2020).

Home oximetry Monitoring Records: For each patient enrolled into a home oximetry monitoring programme at the four sites, data were collected from individual home oximetry monitoring providers consisting of the date of enrolment, oxygen saturations at reast at enrolment and the clinical pathway to which they were enrolled.

Hospital records: The dates, outcomes of Accident and Emergency presentations and admissions to hospital for patients while enrolled in the home oximetry programme were obtained from case note review by participating sites. Data were returned by sites in mid-September 2020.

Mortality records: The date and causes (as ICD-10 codes) of death were provided for each patient whose death had been recorded by the Office for National Statistics after enrolment to the home oximetry programme until the date of data transfer (25th Sept 2020).

Analysis & Statistical Procedure

For peer review only

Data were linked according to the pseudonymised patient identifier. A single cohort of patients was identified where GDPPR data and oxygen saturations at rest at enrolment were available. The demographic and clinical characteristics of this population where described and differences in patient and clinical characteristics were compared between routes of enrolment using pairwise Fisher's Exact Tests for count data and Mann Whitney U-tests for non-parametric continuous data.

The likelihood of a patient presenting to hospital at least once following enrolment to the home oximetry monitoring programme was examined using univariable and multivariable logistic regression models. The likelihood of all-cause mortality following enrolment to the home oximetry monitoring programme was also examined using univariable and multivariable logistic regression models. Frequent absence of dates of enrolment or dates of hospital admission precluded formal temporal evaluation of time to hospital presentation or mortality.

Results

A total of 1338 patients were recorded as being enrolled into the home oximetry programme at one of the four pilot sites. Of these, GDPPR records were available for 1242 patients (92.8%). Of these patients, a recorded oxygen saturation level at enrolment onto the home oximetry was present for 908 patients (73.1%).

Comparison of Enrolment Pathways

The characteristics of the included population, overall and according to the route of enrolment, are described in **Table 1**. 302 patients were enrolled from primary care (33.4%), 342 from A&E (37.9%) and 259 following discharge from hospital (28.9%). Route of enrolment was missing for five patients (0.6%).

Oxygen saturations at enrolment were significantly lower in those enrolled on discharge from hospital (96%) than through A&E (97%) or primary care (98%) (MWU, $p < 0.0001$). No difference was observed between primary care and A&E pathways (MWU, $p = 0.0852$). Similarly, there was no significant difference in the proportion of patients enrolled with oxygen saturations of 95% or more in primary care (92.0%) or A&E (91.8%) (Fishers Exact Test, $p = 1$), however only 67.6% of patients enrolled after hospital discharge had oxygen saturations of 95% or more (pairwise Fishers Exact Tests, $p < 0.0001$).

Patients enrolled through A&E were most likely to have no comorbidities (47.1%) compared to primary care (37.4%) and hospital discharge (27.0). Primary care patients were less likely to have comorbidities than those enrolled after hospital discharge (pairwise Fishers Exact Tests, $p < 0.05$ in all cases). Patients enrolled through A&E were younger (median age = 50 years) than those enrolled in primary care (55 years) and on hospital discharge (63 years) (MWU, $p < 0.0001$ in all cases).

Presentation to Hospital

A total of 69 presentations to hospital were made by 52 patients (5.7%) after enrolment. 40 of these patients (76.9%) were admitted to hospital, and 8 patients (15.4%) presented more than

For peer review only - http://bmjopen.bmj.com/site/about/guidelines.xhtml

once. The proportion of patients presenting to hospital according to age group and number of clinical comorbidities is shown in **Figure 1** and **Figure 2**.

3.1% of patients under the age of 65 years and without comorbidities presented to hospital, compared to 5% of those under 65 with comorbidities and 9.7% of those aged 65 and over. Similarly, 5.3% of patients with oxygen saturations of 95% or more at enrolment presented to hospital, compared to 6.1% of those with oxygen saturations of 93-94% and 10.9% of those with oxygen saturations less than 93% at enrolment (**Table 2**).

In univariable logistic regression models, presentation to hospital was significantly more likely in older patients and those with more clinical comorbidities, while those initiated on the home oximetry through A&E or on discharge from hospital were less likely to re-present to hospital (**Table 3**). In the multivariable model, increasing age (OR 1.03, p = 0.018) was associated with significantly higher odds of presentation to hospital, while those initiated through A&E (OR 0.42, p = 0.024) and following discharge from hospital (OR 0.31, p = 0.003) were significantly less likely to present to hospital (**Table 3**).

All-cause mortality

A total of 28 patients (3.1%) died of any cause following enrolment. 14 of these patients (50%) had Covid-19 as a named cause of death (ICD-10 code U07.1), and 12 (42.9%) had Covid-19 as the underlying cause of death. All-cause mortality by age group and number of clinical comorbidities is shown in **Figure 1** and **Figure 2**. Only patients with one or more comorbidities died, while 17 (60.1%) patients who died had four or more clinical comorbidities.

None of the 320 patients under the age of 65 and without comorbidities included in the study died during the study period, compared to 0.9% of those under 65 with comorbidities and 9.3% of those aged 65 and over. 2.6% of patients with oxygen saturations of 95% or more at enrolment died during the study period, compared to 6.1% of those with oxygen saturations of 93-94% and 5.5% of those with oxygen saturations less than 93% at enrolment (**Table 2**).

In univariable logistic regression models, all-cause mortality was significantly more likely in older patients, those of BAME ethnicity, with more clinical comorbidities, who were overweight or obese and those initiated on the home oximetry following discharge from hospital (**Table 4**). In the multivariable model, increasing age (OR 1.08, p = 0.000), more clinical comorbidities (OR 1.45, p = 0.009), being overweight or obese (OR 4.83, p = 0.002) and being initiated on the home oximetry following discharge from hospital (OR 8.70, p = 0.001) were associated with significantly higher odds of all-cause mortality (**Table 4**).

Discussion

Statement of key findings

In terms of clinical safety, only 5.7% of patients presented to hospital after enrolment. The likelihood of presenting to hospital increased with age, but was not significantly associated

For peer review only - http://bmjopen.bmj.com/site/about/guidelines.xhtml

with ethnicity, number of clinical comorbidities, obesity or oxygen saturations at enrolment. However, all-cause mortality was significantly more likely with increasing age, number of clinical comorbidities and obesity. All-cause mortality was also higher for those patients who initiated the pathway after discharge from hospital. Finally, most patients using home oximetry monitoring were of low clinical severity on initiation and neither died nor presented to hospital during the study period. Therefore, the Covid-19 assessment pathway published in June 2020 is clinically appropriate, and its recommendations regarding oxygen saturation thresholds are correct.

While hospital presentation and mortality were associated with lower oxygen saturations at enrolment, this was not a statistically significant relationship after controlling for other patient factors. Therefore, although oxygen saturation at enrolment may provide an important tool by which to stratify clinical severity, such assessments must also incorporate a wider understanding of factors determining outcome including age, obesity and clinical comorbidities.

None of the 320 participants aged less than 65 and without comorbidities died during the study period. These individuals accounted for 33.3% of all participants which suggests that during the pilots a large proportion of the participants were low risk at enrolment and did not suffer any adverse outcome. Wider implementation should therefore focus on higher risk groups to ensure efficient use of limited health and care resources.

Statistically significant variation in the characteristics of patients, clinical severity of illness, rates of hospital presentation and all-cause mortality were observed between the three routes of enrolment. Patients enrolled following a hospital admission had 8.7 times higher odds of all cause mortality than patients enrolled in primary care after adjusting for age, sex, ethnicity, obesity, clinical comorbidities and oxygen saturation at enrolment. Conversely, compared to those enrolled in primary care, patients enrolled after an A&E presentation or hospital admission had significantly lower odds of further presentation to hospital after enrolment. Collectively, this indicates differences in patient characteristics and the risk of adverse events according to the route of enrolment. Those enrolled in primary care are generally the least unwell and go on to have lower rates of hospital presentation and mortality. The finding that patients enrolled in A&E or following hospital admission have lower rates of hospital presentation may indicate reluctance on the part of patients to return to hospital during the same period of illness. Patients enrolled following hospital discharge appear to be at particularly high risk of mortality and may therefore represent a patient population that should either be offered more intensive monitoring or alternative approaches to reducing the risk of subsequent mortality.

Following enrolment more patients aged 90 years and over died during the study period than presented to hospital, while in younger age groups hospital presentations outnumbered all-cause mortality (Figure 1). A similar trend was observed according to increasing clinical comorbidities (Figure 2). This is likely to reflect circumstances in which hospital admission is not in accordance with a patient's wishes or considered clinically appropriate, and end-of-life care is therefore initiated in a person's usual place of residence.

For peer review only - http://bmjopen.bmj.com/site/about/guidelines.xhtml

Taken together, our results support the hypothesis that oximetry monitoring is a safe pathway for Covid-19 patients. This was reported to NHS England and Improvement in October 2020.

Strengths & Limitations

The primary strength of this study is its system-level and patient-level impact, since this work evaluated a real-world pilot of a new clinical pathway. The project team achieved an integrated partnership between academics, clinicians and policy makers. This enabled a direct pipeline from evidence generation to policy decision making; moreover, the efficiency with which the work was conducted was nationally important, as it was required to inform how the NHS would use home oximetry monitoring during the next wave of the pandemic. Impact was achieved through the unique composition of the evaluation team as well as the close relationship the team held to NHS leaders in home oximetry monitoring. The method for this work required novel data reporting from the pilot sites to the evaluation team and this was ensured through close collaboration with the individual sites.

The most prevalent limitation related to data quality and completeness. The pilot sites varied in their routes of referral and in the clinical severity of the patients they enrolled. Furthermore, there was heterogeneity in the actual intervention across the sites, as some enrolled patients with suspected Covid-19, while others included patients who had received hospital treatment for Covid-19 and were subsequently discharged. Due to the rapid timescales of this evaluation and the pressured environment in which it took place, mandating full completeness in the data submitted was not possible, and the resulting dataset had a considerable amount of missing data. In addition, the absence of precise temporal recording of data and an inability to complete individual case note reviews precluded a more detailed evaluation of whether deaths occurred as a result of Covid-19 infection, or another cause entirely. Finally, this was not a controlled study, which prevented a comparative analysis. Due to these limitations and the relatively low community prevalence of Covid-19 during the pilot, our findings are not necessarily generalisable to future waves of Covid-19.

Comparison to other studies

While research evidence regarding Covid-19 home oximetry is still in its infancy, this study reflects similar findings in the published literature. One systematic review of home monitoring for Covid-19 acknowledges inconclusive evidence about effectiveness and safety. While this study could not conclusively determine the clinical effectiveness of the pilots, it was able to demonstrate non-inferiority in terms of safety with traditional hospital management of Covid-19 (15). Furthermore, this study indicates the potential for these technologies to support pandemic management in line with expert opinions published in the literature (16). The findings of this work are consistent with existing grey literature suggesting the utility of home oximetry monitoring for Covid-19 (8)(17). This study specifically assessed the safety of home oximetry for Covid-19 and makes policy recommendations not present in existing studies which tend to focus more on patient experience and potential clinical effectiveness.

Implications for Policy

This work was explicitly designed to answer relevant questions in advance of the national implementation of a new clinical pathway for Covid-19. It confirms the appropriateness of existing policies and that safety netting approaches are congruent with findings.

For peer review only - http://bmjopen.bmj.com/site/about/guidelines.xhtml

This study provides information as to how the pathway should function including the thresholds at which people should be enrolled into home oximetry monitoring programmes, how long they should continue in home oximetry monitoring and what sorts of technology is required to support home oximetry monitoring for Covid-19.

Furthermore, the practicalities of running this study revealed important considerations for the ongoing evaluation of home oximetry monitoring pathways for Covid-19. It is clear from the study that it is only possible to measure the safety of home oximetry monitoring with the robust and rigorous collection of data from healthcare providers as well as any third party app-based providers. This data flow should continue during national implementation to continuously measure safety under different conditions and levels of organisational pressure.

Conclusion

Advancing the evidence base regarding the safety of home oximetry monitoring for Covid-19 patients is of considerable importance as many health systems face new waves of the pandemic worldwide. This study reveals, via a real-world pilot evaluation, that home oximetry monitoring can be a safe pathway for Covid patients; however, substantial research is needed to understand its clinical effectiveness across patient populations. This study was limited by complex and incomplete data as well as variation in intervention designs; a product of the pandemic context within which it was undertaken. However, it did provide initial evidence of the appropriate clinical thresholds and patient characteristics for home oximetry. It also indicated increases in risk to vulnerable groups and patients with oxygen saturations below 95% at enrolment.

Findings from this evaluation have contributed to the national implementation of home oximetry across England, and further work will be undertaken to evaluate clinical effectiveness and any inequalities in terms of access to, and inclusion in, the new pathway.

For peer review only

For peer review only - http://bmjopen.bmj.com/site/about/guidelines.xhtml

Funding

This research was supported by the National Institute for Health Research (NIHR) Imperial Patient Safety Translational Research Centre (PSTRC). The views expressed are those of the authors and not necessarily those of the NHS, the NIHR or the Department of Health and Social Care. JC acknowledges support from the Wellcome Trust (215938/Z/19/Z).

Conflicts of Interest

All authors have completed the ICMJE uniform disclosure form at www.icmje.org/coi_disclosure.pdf and have no competing interests to declare.

Acknowledgements

The research team would like to thank the teams at NHS Digital and NHS England/Improvement as well as the site leads from the home oximetry pilot sites.

Data Sharing Statement

Patient-level pseudonymised data were obtained from NHS Digital for the specific purpose of the evaluation. Aggregate findings are presented in this study. Patient-level data may be obtained through application and review by NHS Digital.

Contribution Statement

The corresponding author attests that all listed authors meet authorship criteria and that no others meeting the criteria have been omitted.

Copyright Statement

The Corresponding Author has the right to grant on behalf of all authors and does grant on behalf of all authors, a worldwide licence to the Publishers and its licensees in perpetuity, in all forms, formats and media (whether known now or created in the future), to i) publish, reproduce, distribute, display and store the Contribution, ii) translate the Contribution into other languages, create adaptations, reprints, include within collections and create summaries, extracts and/or, abstracts of the Contribution, iii) create any other derivative work(s) based on the Contribution, iv) to exploit all subsidiary rights in the Contribution, v) the inclusion of electronic links from the Contribution to third party material where-ever it may be located; and, vi) licence any third party to do any or all of the above.

Patient and Public Involvement Statement

Patients and/or the public were not involved in the design, or conduct, or reporting, or dissemination plans of this research.

For peer review only

Transparency Declaration

JC affirms that the manuscript is an honest, accurate, and transparent account of the study being reported; that no important aspects of the study have been omitted; and that any discrepancies from the study as originally planned (and, if relevant, registered) have been explained.

Ethics Approval Statement

The work was conducted as a service evaluation, as institutional the Imperial College Research Ethics Committee deemed that it did not require further ethics committee approval.

For peer review only

Citations

1. Greenhalgh T, Vijayaraghavan S, Wherton J, Shaw S, Byrne E, Campbell-Richards D, et al. Virtual online consultations: advantages and limitations (VOCAL) study. BMJ Open. 2016 Jan 1;6(1):e009388.

2. National Information Board. Personalised Health and Care 2020 Using Data and Technology to Transform Outcomes for Patients and Citizens A Framework for Action [Internet]. National Information Board; 2014 Nov. Available from: https://www.gov.uk/government/uploads/system/uploads/attachment_data/file/384650/NIB_Report.pdf

3. Joshi M, Ashrafian H, Arora S, Khan S, Cooke G, Darzi A. Digital Alerting and Outcomes in Patients With Sepsis: Systematic Review and Meta-Analysis. J Med Internet Res. 2019;21(12):e15166.

4. Noah B, Keller MS, Mosadeghi S, Stein L, Johl S, Delshad S, et al. Impact of remote patient monitoring on clinical outcomes: an updated meta-analysis of randomized controlled trials. Npj Digit Med. 2018 Jan 15;1(1):1–12.

5. Kim Y-N, Shin DG, Park S, Lee CH. Randomized clinical trial to assess the effectiveness of remote patient monitoring and physician care in reducing office blood pressure. Hypertens Res Off J Jpn Soc Hypertens. 2015 Jul;38(7):491–7.

6. Design of the Pilot, Proof of Concept REMOTE-COVID Trial: Remote Monitoring Use in Suspected Cases of COVID-19 (SARS-CoV 2) A Trial Protocol. 2020 Sep 22 [cited 2020 Dec 14]; Available from: https://www.researchsquare.com/article/rs-78141/v1

7. Medina M, Babiuch C, Card M, Gavrilescu R, Zafirau W, Boose E, et al. Home monitoring for COVID-19. Cleve Clin J Med [Internet]. 2020 May 14 [cited 2020 Oct 30]; Available from: https://www.ccjm.org/content/early/2020/06/08/ccjm.87a.ccc028

8. Coronavirus » Pulse oximetry to detect early deterioration of patients with COVID-19 in primary and community care settings [Internet]. [cited 2020 Oct 30]. Available from: https://www.england.nhs.uk/coronavirus/publication/pulse-oximetry-to-detect-early-deterioration-of-patients-with-covid-19-in-primary-and-community-care-settings/

9. Gerke S, Shachar C, Chai PR, Cohen IG. Regulatory, safety, and privacy concerns of home monitoring technologies during COVID-19. Nat Med. 2020 Aug;26(8):1176–82.

10. Morgan AU, Balachandran M, Do D, Lam D, Parambath A, Chaiyachati KH, et al. Remote Monitoring of Patients with Covid-19: Design, implementation, and outcomes of the first 3,000 patients in COVID Watch. Nejm Catal Innov Care Deliv [Internet]. 2020 Jul 21 [cited 2020 Oct 30]; Available from: https://www.ncbi.nlm.nih.gov/pmc/articles/PMC7380701/

11. Taiwo O, Ezugwu AE. Smart healthcare support for remote patient monitoring during covid-19 quarantine. Inform Med Unlocked. 2020;20:100428.

12. Watson AR, Wah R, Thamman R. The Value of Remote Monitoring for the COVID-19 Pandemic. Telemed E-Health. 2020 May 7;26(9):1110–2.

13. Levitan RM. Pulse Oximetry as a Biomarker for Early Identification and Hospitalization of COVID-19 Pneumonia. Acad Emerg Med. 2020;27(8):785–6.

For peer review only

14. Luks AM, Swenson ER. Pulse Oximetry for Monitoring Patients with COVID-19 at Home. Potential Pitfalls and Practical Guidance. Ann Am Thorac Soc. 2020 Jun 10;17(9):1040–6.

15. Vindrola-Padros C, Singh KE, Sidhu MS, Georghiou T, Sherlaw-Johnson C, Tomini SM, et al. Remote home monitoring (virtual wards) during the COVID-19 pandemic: a living systematic review. medRxiv. 2020 Oct 9;2020.10.07.20208587.

16. Zhao M, Wasfy JH, Singh JP. Sensor-aided continuous care and self-management: implications for the post-COVID era. Lancet Digit Health [Internet]. 2020 Sep 16 [cited 2020 Oct 30];0(0). Available from: https://www.thelancet.com/journals/landig/article/PIIS2589-7500(20)30220-X/abstract

17. COVID-19 Virtual Wards Data [Internet]. NHS Digital. [cited 2020 Oct 31]. Available from: https://digital.nhs.uk/about-nhs-digital/corporate-information-and-documents/directions-and-data-provision-notices/data-provision-notices-dpns/covid-19-virtual-wards-data

For peer review only

Figures:

Figure 1: Percentage all-cause mortality and presentation to hospital according to patient age

Figure 2: Percentage all-cause mortality and presentation to hospital according to number of clinical comorbidities

For peer review only

For peer review only - http://bmjopen.bmj.com/site/about/guidelines.xhtml

Tables:

Table 1: Characteristics of the study population, overall and according to the route of enrolment. NB: Route of enrolment was missing for 5 of 908 patients.

		Primary Care	Accident & Emergency	Inpatient discharge	Overall
Total Patients		302	342	259	908
Female		180 (59.6)	205 (59.9)	120 (46.3)	508 (55.9)
Median Age (years)		55	50	63	54
Ethnicity	White	113 (37.4)	160 (46.8)	140 (54.1)	415 (45.7)
	Black, Asian or Minority Ethnic	82 (27.2)	103 (30.1)	59 (22.8)	244 (26.9)
	Not recorded	107 (35.4)	79 (23.1)	60 (23.2)	249 (27.4)
Overweight or obese		108 (35.8)	189 (55.3)	160 (61.8)	458 (50.4)
Median oxygen saturations		98	97	96	97
Clinical severity based on oxygen saturations	Mild (≥ 95%)	278 (92.1)	314 (91.8)	175 (67.6)	771 (84.9)
	Moderate (93-94%)	17 (5.6)	22 (6.4)	42 (16.2)	82 (9.0)
	Severe (≤ 92%)	7 (2.3)	6 (1.8)	42 (16.2)	55 (6.1)
Number of clinical comorbidities	0	113 (37.4)	161 (47.1)	70 (27.0)	346 (38.1)
	1	75 (24.8)	105 (30.7)	70 (27.0)	252 (27.7)
	2	42 (13.9)	45 (13.2)	44 (17.0)	131 (14.4)
	3+	72 (23.8)	31 (9.1)	75 (29.0)	179 (19.7)

Table 2: Number of patients, frequency of hospital presentation and all-cause mortality according to risk groups and oxygen saturations at enrolment

Outcome	Risk Group	Oxygen Saturations Severity			
		Mild (≥ 95%)	Moderate (93-94%)	Severe (≤ 92%)	Overall
Total Patients	>= 65 years	203 (75.5)	37 (13.8)	29 (10.8)	269 (29.6)
	< 65 years with comorbidities	277 (86.8)	29 (9.1)	13 (4.1)	319 (35.1)
	< 65 years without comorbidities	291 (90.9)	16 (5.0)	13 (4.1)	320 (35.2)
	Overall	771 (84.9)	82 (9.0)	55 (6.1)	908 (100.0)
Hospital presentation	>= 65 years	18 (8.9)	3 (8.1)	5 (17.2)	26 (9.7)
	< 65 years with comorbidities	13 (4.7)	2 (6.9)	1 (7.7)	16 (5.0)
	< 65 years without comorbidities	10 (3.4)	0 (0.0)	0 (0.0)	10 (3.1)
	Overall	41 (5.3)	5 (6.1)	6 (10.9)	52 (5.7)
All-cause mortality	>= 65 years	18 (8.9)	4 (10.8)	3 (10.3)	25 (9.3)
	< 65 years with comorbidities	2 (0.7)	1 (3.4)	0 (0.0)	3 (0.9)
	< 65 years without comorbidities	0 (0.0)	0 (0.0)	0 (0.0)	0 (0.0)
	Overall	20 (2.6)	5 (6.1)	3 (5.5)	28 (3.1)

For peer review only - http://bmjopen.bmj.com/site/about/guidelines.xhtml

Table 3: Univariable and multivariable binary logistic regression output for presentation to hospital following enrolment to the home oximetry pathway

		Univariable			Multivariable		
		OR	p-value	95% CI of OR	OR	p-value	95% CI of OR
Age		1.03	**0.000**	1.01 - 1.05	1.03	**0.018**	1.00 - 1.05
Ethnicity	White	reference			reference		
	Black, Asian and Minority Ethnic	1.22	0.544	0.64 - 2.32	1.57	0.198	0.79 - 3.10
	Not recorded	0.75	0.447	0.36 - 1.57	0.95	0.912	0.42 - 2.17
Number of comorbidities		1.25	**0.004**	1.08 - 1.45	1.07	0.503	0.88 - 1.30
Overweight or Obese		0.67	0.175	0.38 - 1.19	0.55	0.069	0.29 - 1.05
Clinical severity based on oxygen saturations	Mild (≥ 95%)	reference			reference		
	Moderate (93-94%)	1.16	0.766	0.44 - 3.01	1.08	0.885	0.40 - 2.92
	Severe (≤ 92%)	2.18	0.091	0.88 - 5.39	2.35	0.096	0.86 - 6.46
Enrolment Pathway	Primary care	reference			reference		
	Accident & Emergency	0.37	**0.005**	0.18 - 0.74	0.42	**0.024**	0.20 - 0.89
	Inpatient	0.49	**0.049**	0.25 - 0.98	0.31	**0.003**	0.15 - 0.68

Table 4: Univariable and multivariable binary logistic regression output for all-cause mortality following enrolment to the home oximetry pathway

		Univariable			Multivariable		
		OR	p	95% CI of OR	OR	p	95% CI of OR
Age		1.12	**0.000**	1.08 - 1.16	1.08	**0.000**	1.03 - 1.13
Ethnicity	White	reference			reference		
	Black, Asian and Minority Ethnic	0.16	**0.015**	0.04 - 0.70	0.37	0.235	0.07 - 1.90
	Not recorded	0.49	0.129	0.19 - 1.23	0.61	0.371	0.21 - 1.79
Number of comorbidities		1.97	**0.000**	1.63 - 2.40	1.45	**0.009**	1.09 - 1.92
Overweight or Obese		3.87	**0.004**	1.56 - 9.64	4.83	**0.002**	1.74 - 13.37
Clinical severity based on oxygen saturations	Mild (≥ 95%)	reference			reference		
	Moderate (93-94%)	2.43	0.083	0.89 - 6.68	1.11	0.864	0.34 - 3.67
	Severe (≤ 92%)	2.16	0.224	0.62 - 7.53	0.65	0.574	0.14 - 2.93
Enrolment Pathway	Primary care	reference			reference		
	Accident &Emergency	0.66	0.587	0.15 - 2.97	3.40	0.157	0.62 - 18.55
	Inpatient	6.57	**0.001**	2.23 - 19.41	8.70	**0.001**	2.53 - 29.89

For peer review only - http://bmjopen.bmj.com/site/about/guidelines.xhtml

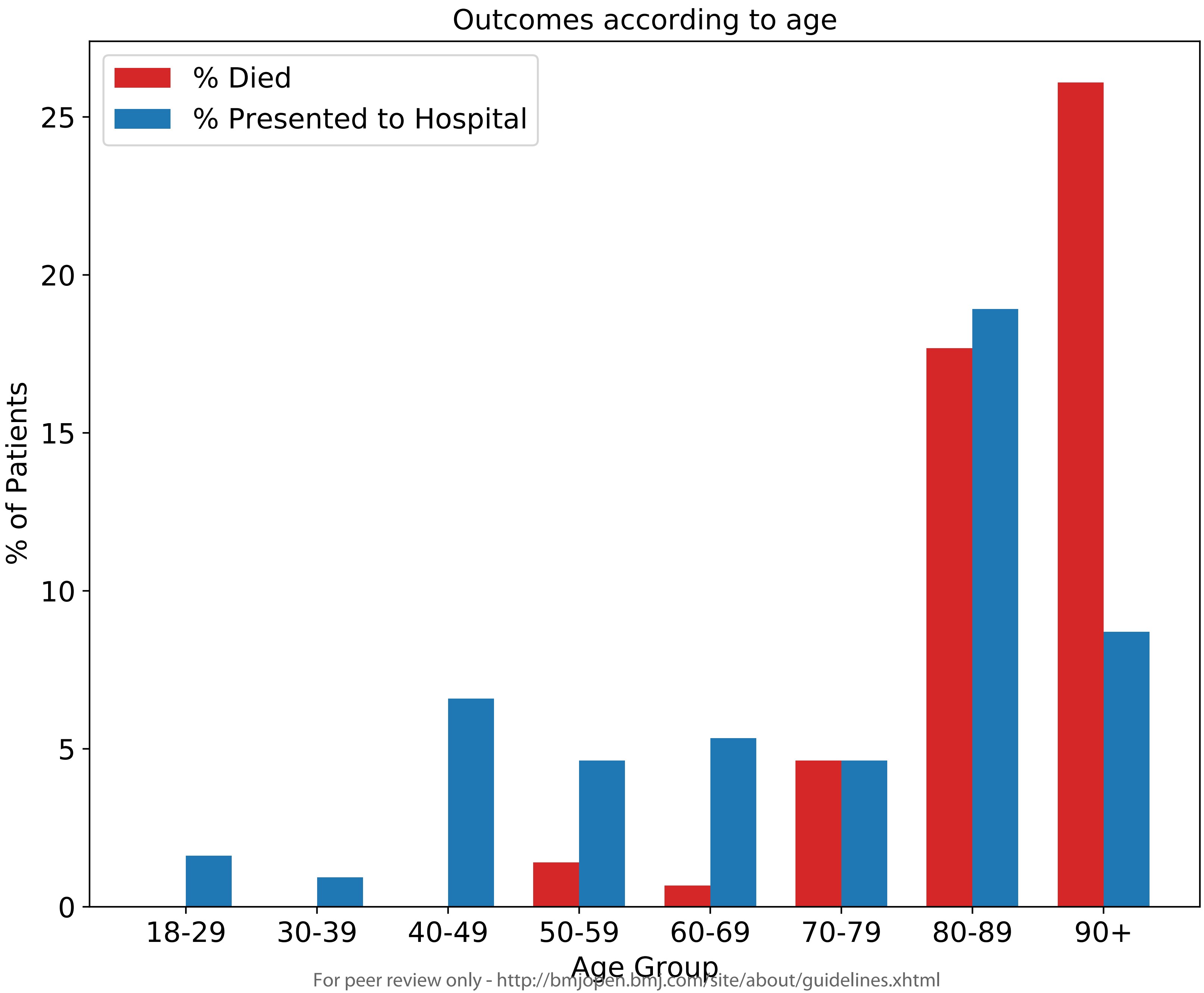
Outcomes according to age
% Died
% Presented to Hospital
% of Patients
0
5
10
15
20
25
18-29
30-39
40-49
50-59
60-69
70-79
80-89
90+
Age Group

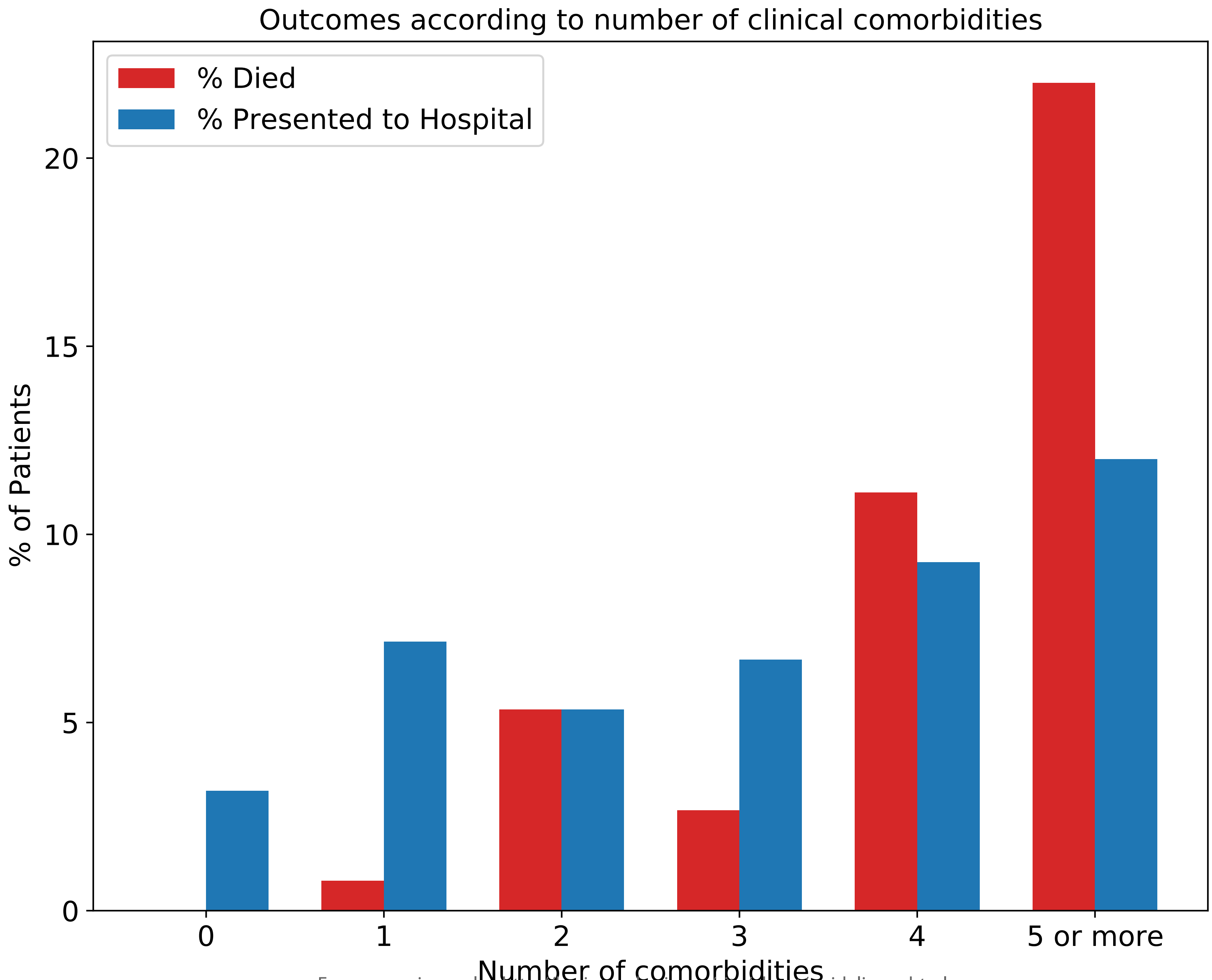
Outcomes according to number of clinical comorbidities
% Died
% Presented to Hospital
% of Patients
0
5
10
15
20
0
1
2
3
4
5 or more
Number of comorbidities

Category	Variable
Baseline Data	Age
	Ethnicity
	Sex
	post code
	occupation
	Registered GP Practice
	Home circumstances
	Smoker
	**Clinical Comorbidities:***
	BMI
	Asthma
	COPD
	Emphysema
	Bronchiectasis
	Other Chronic Resp Disease
	Chronic cardiac disease
	Chronic liver disease
	Stroke or dementia
	Neurological
	Chronic kidney disease
	Hypertension
	Diabetes
	Cancer
	Cancer site(s) / types
	Date of Cancer Diagnosis
	HIV
	Transplant receipient
	Transplant sites
	Immunosuppression regimen
Initial Entry	**Onset of Symptoms:**
	Breathlessness
	Myalgia
	Chills
	Severe fatigue
	Sputum
	Dizziness
	Cough
	Nausea or vomiting
	Diarrhoea
	Headache
	Sore throat
	Nasal congestion
	Anosmia
	Unwell contact
	Sars COV 2 swab positive
	Referral source

	Date and time of recording
	**Clinical observations:**
	O2 sats at rest
	O2 sats after exertion
	Heart rate
	Respiratory rate
	Blood pressure
	Conscious level (GCS)
	Temperature
	NEWS Score

Diary Entry (iterates 2-3x daily)	Date and time of recording
	**Clinical observations:**
	O2 sats at rest
	O2 sats after exertion
	HR
	Temperature

Diary Aggregate Data (N.B. Derived from Diary entries - not collected in itself)	*Mean pulse*
	Mean O2 sats
	Datetime of lowest O2 Sats
	Value of lowest O2 Sats
	Datetime of highest HR
	Value of highest HR
	Datetime of highest Temp
	Value of highest Temp
	Number of days of fever
	Days on Virtual Ward

In Hospital Data (one for each A&E presentation or hospital admission)	First location on arrival
	Date and time of arrival
	Admitted from A&E
	**Obs on arrival:**
	O2 sats on air
	O2 desturation (>3%)
	Heart rate
	Respiratory rate
	Blood pressure
	Conscious level (GCS)
	Temperature
	NEWS Score
	**Treatment given:**
	Highest Oxygen Requirement

	High flow oxygen
	CPAP
	NIV
	Intubation
	level 2 care
	Level 3 care (ITU)
	**Outcomes:**
	Date and time of discharge or in-hospital de
	Discharge destination
	Length of Stay

Mortality	Date of Death
	Cause(s) of Death

For peer review only

Source	Data Type
PDS	Numeric
PDS	Categorical
PDS	Categorical
PDS	String
Onboarding	Categorical /string
PDS	String
Onboarding	Categorical
Onboarding / GP	Categorical
Onboarding / GP	Numeric
Onboarding / GP	Binary
Onboarding / GP	Binary
Onboarding / GP	Binary
Onboarding / GP	Binary
Onboarding / GP	Binary / string
Onboarding / GP	Binary
Onboarding / GP	Binary
Onboarding / GP	Binary
Onboarding / GP	Binary
Onboarding / GP	Binary
Onboarding / GP	Binary
Onboarding / GP	Binary
Onboarding / GP	Binary
Onboarding / GP	String
Onboarding / GP	Date
Onboarding / GP	Binary
Onboarding / GP	Binary
Onboarding / GP	Cateogrical
Onboarding / GP	String
Onboarding	Date
Onboarding	Date
Onboarding	Date
Onboarding	Date
Onboarding	Date
Onboarding	Date
Onboarding	Date
Onboarding	Date
Onboarding	Date
Onboarding	Date
Onboarding	Date
Onboarding	Date
Onboarding	Date
Onboarding	Binary
Onboarding	Categorical
Onboarding	Categorical

For peer review only - http://bmjopen.bmj.com/site/about/guidelines.xhtml

Onboarding	Timestamp
Onboarding	Numerical
Onboarding	Numerical
Onboarding	Numerical
Onboarding	Numerical
Onboarding	Numerical
Onboarding	Numerical
Onboarding	Numerical
Onboarding	Numerical

Diary	Timestamp
Diary	Numerical
Diary	Numerical
Diary	Numerical
Diary	Numerical

Calculated from Diary	*Numeric*
Calculated from Diary	*Numeric*
Calculated from Diary	*Numeric*
Calculated from Diary	*Numeric*
Calculated from Diary	*Numeric*
Calculated from Diary	*Numeric*
Calculated from Diary	*Numeric*
Calculated from Diary	*Numeric*
Calculated from Diary	*Numeric*
Calculated from Diary	*Numeric*

Hospital EHR / HES	Categorical
Hospital EHR / HES	Timestamp
Hospital EHR / HES	Binary
Hospital EHR	Numeric
Hospital EHR	Numeric
Hospital EHR	Numeric
Hospital EHR	Numeric
Hospital EHR	Numeric
Hospital EHR	Numeric
Hospital EHR	Numeric
Hospital EHR	Numeric
Hospital EHR	Numeric

Hospital EHR	Binary
Hospital EHR	Binary
Hospital EHR	Binary
Hospital EHR	Binary
Hospital EHR	Binary
Hospital EHR	Binary
Hospital EHR / HES	Timestamp
Hospital EHR / HES	Categorical
Computed from in-hospital data	*Numeric*

ONS	Date
ONS	String

For peer review only

STROBE Statement—checklist of items that should be included in reports of observational studies

	Item No	Recommendation	Page No
**Title and abstract**	1	(*a*) Indicate the study's design with a commonly used term in the title or the abstract	2
		(*b*) Provide in the abstract an informative and balanced summary of what was done and what was found	2
**Introduction**			
Background/rationale	2	Explain the scientific background and rationale for the investigation being reported	3
Objectives	3	State specific objectives, including any prespecified hypotheses	3
**Methods**			
Study design	4	Present key elements of study design early in the paper	4
Setting	5	Describe the setting, locations, and relevant dates, including periods of recruitment, exposure, follow-up, and data collection	4
Participants	6	(*a*) *Cohort study*—Give the eligibility criteria, and the sources and methods of selection of participants. Describe methods of follow-up *Case-control study*—Give the eligibility criteria, and the sources and methods of case ascertainment and control selection. Give the rationale for the choice of cases and controls *Cross-sectional study*—Give the eligibility criteria, and the sources and methods of selection of participants	4
		(*b*) *Cohort study*—For matched studies, give matching criteria and number of exposed and unexposed *Case-control study*—For matched studies, give matching criteria and the number of controls per case	N/A
Variables	7	Clearly define all outcomes, exposures, predictors, potential confounders, and effect modifiers. Give diagnostic criteria, if applicable	4
Data sources/ measurement	8*	For each variable of interest, give sources of data and details of methods of assessment (measurement). Describe comparability of assessment methods if there is more than one group	*4*
Bias	9	Describe any efforts to address potential sources of bias	4,5
Study size	10	Explain how the study size was arrived at	4
Quantitative variables	11	Explain how quantitative variables were handled in the analyses. If applicable, describe which groupings were chosen and why	4,5
Statistical methods	12	(*a*) Describe all statistical methods, including those used to control for confounding	5
		(*b*) Describe any methods used to examine subgroups and interactions	5
		(*c*) Explain how missing data were addressed	5
		(*d*) *Cohort study*—If applicable, explain how loss to follow-up was addressed *Case-control study*—If applicable, explain how matching of cases and controls was addressed *Cross-sectional study*—If applicable, describe analytical methods taking account of sampling strategy	N/A
		(*e*) Describe any sensitivity analyses	N/A

Continued on next page

For peer review only - http://bmjopen.bmj.com/site/about/guidelines.xhtml

**Results**			
Participants	13*	(a) Report numbers of individuals at each stage of study—eg numbers potentially eligible, examined for eligibility, confirmed eligible, included in the study, completing follow-up, and analysed	5
		(b) Give reasons for non-participation at each stage	5
		(c) Consider use of a flow diagram	N/A
Descriptive data	14*	(a) Give characteristics of study participants (eg demographic, clinical, social) and information on exposures and potential confounders	5,14
		(b) Indicate number of participants with missing data for each variable of interest	5
		(c) *Cohort study*—Summarise follow-up time (eg, average and total amount)	N/A
Outcome data	15*	*Cohort study*—Report numbers of outcome events or summary measures over time	
		Case-control study—Report numbers in each exposure category, or summary measures of exposure	
		Cross-sectional study—Report numbers of outcome events or summary measures	*6*
Main results	16	(*a*) Give unadjusted estimates and, if applicable, confounder-adjusted estimates and their precision (eg, 95% confidence interval). Make clear which confounders were adjusted for and why they were included	^
		(*b*) Report category boundaries when continuous variables were categorized	N/A
		(*c*) If relevant, consider translating estimates of relative risk into absolute risk for a meaningful time period	N/A
Other analyses	17	Report other analyses done—eg analyses of subgroups and interactions, and sensitivity analyses	5,6
**Discussion**			
Key results	18	Summarise key results with reference to study objectives	7
Limitations	19	Discuss limitations of the study, taking into account sources of potential bias or imprecision. Discuss both direction and magnitude of any potential bias	8
Interpretation	20	Give a cautious overall interpretation of results considering objectives, limitations, multiplicity of analyses, results from similar studies, and other relevant evidence	7-9
Generalisability	21	Discuss the generalisability (external validity) of the study results	8-9
**Other information**			
Funding	22	Give the source of funding and the role of the funders for the present study and, if applicable, for the original study on which the present article is based	10

*Give information separately for cases and controls in case-control studies and, if applicable, for exposed and unexposed groups in cohort and cross-sectional studies.

Note: An Explanation and Elaboration article discusses each checklist item and gives methodological background and published examples of transparent reporting. The STROBE checklist is best used in conjunction with this article (freely available on the Web sites of PLoS Medicine at http://www.plosmedicine.org/, Annals of Internal Medicine at http://www.annals.org/, and Epidemiology at http://www.epidem.com/). Information on the STROBE Initiative is available at www.strobe-statement.org.

For peer review only

For peer review only - http://bmjopen.bmj.com/site/about/guidelines.xhtml

BMJ Open

Assessing the Safety of Home Oximetry for Covid-19: A multi-site retrospective observational study.

Journal:	*BMJ Open*
Manuscript ID	bmjopen-2021-049235.R1
Article Type:	Original research
Date Submitted by the Author:	19-May-2021
Complete List of Authors:	Clarke, Jonathan; Imperial College London, Centre for Mathematics of Precision Healthcare<br>Flott, Kelsey; Imperial College London, Institute of Global Health Innovation<br>Fernandez Crespo, Roberto; Imperial College London, Institute of Global Health Innovation<br>Ashrafian, Hutan; Imperial College London, Institute of Global Health Innovation<br>Fontana, Gianluca; Imperial College London, Institute of Global Health Innovation<br>Benger, Jonathan; NHS Digital<br>Darzi, Ara; Imperial College London, Institute of Global Health Innovation<br>Elkin, Sarah; Imperial College London, National Heart and Lung Institute
**Primary Subject Heading**:	Respiratory medicine
Secondary Subject Heading:	Diagnostics, General practice / Family practice, Public health, Infectious diseases, Health services research
Keywords:	COVID-19, Telemedicine < BIOTECHNOLOGY & BIOINFORMATICS, PRIMARY CARE, PUBLIC HEALTH, RESPIRATORY MEDICINE (see Thoracic Medicine)

SCHOLARONE™
Manuscripts

I, the Submitting Author has the right to grant and does grant on behalf of all authors of the Work (as defined in the below author licence), an exclusive licence and/or a non-exclusive licence for contributions from authors who are: i) UK Crown employees; ii) where BMJ has agreed a CC-BY licence shall apply, and/or iii) in accordance with the terms applicable for US Federal Government officers or employees acting as part of their official duties; on a worldwide, perpetual, irrevocable, royalty-free basis to BMJ Publishing Group Ltd ("BMJ") its licensees and where the relevant Journal is co-owned by BMJ to the co-owners of the Journal, to publish the Work in this journal and any other BMJ products and to exploit all rights, as set out in our licence.

The Submitting Author accepts and understands that any supply made under these terms is made by BMJ to the Submitting Author unless you are acting as an employee on behalf of your employer or a postgraduate student of an affiliated institution which is paying any applicable article publishing charge ("APC") for Open Access articles. Where the Submitting Author wishes to make the Work available on an Open Access basis (and intends to pay the relevant APC), the terms of reuse of such Open Access shall be governed by a Creative Commons licence – details of these licences and which Creative Commons licence will apply to this Work are set out in our licence referred to above.

Other than as permitted in any relevant BMJ Author's Self Archiving Policies, I confirm this Work has not been accepted for publication elsewhere, is not being considered for publication elsewhere and does not duplicate material already published. I confirm all authors consent to publication of this Work and authorise the granting of this licence.

For peer review only

Assessing the Safety of Home Oximetry for Covid-19: A multi-site retrospective observational study.

Authors:

Jonathan Clarke[1]

Kelsey Flott[2]

Roberto Crespo[2]

Hutan Ashrafian[2]

Gianluca Fontana[2]

Jonathan Benger[3]

Ara Darzi[2]

Sarah Elkin[4]

Institution:

1. Centre for Mathematics of Precision Healthcare, Imperial College London, London, SW7 2AZ
2. Institute of Global Health Innovation, Imperial College London, London, SW7 2NA
3. NHS Digital, 1 Trevelyan Square, Boar Lane, Leeds, West Yorkshire. LS1 6AE.
4. National Heart and Lung Institute, Imperial College London, Guy Scadding Building, Cale Street, London, SW3 6LY

Word count:

3515

Corresponding Author

Dr Jonathan Clarke

Centre for Mathematics of Precision Healthcare, Imperial College London, London SW7 2AZ

j.clarke@imperial.ac.uk

+44 (0) 7792 690037

For peer review only

Abstract

Objectives

To determine the safety and effectiveness of home oximetry monitoring pathways safe for Covid-19 patients in the English NHS.

Design

This was a retrospective, multi-site, observational study of home oximetry monitoring for patients with suspected or proven Covid-19 Setting This study analysed patient data from four Covid-19 home oximetry pilot sites in England across primary and secondary care settings.

Participants

1338 participants were enrolled in a home oximetry programme at one of the four pilot sites. Participants were excluded if primary care data and oxygen saturations are rest at enrolment were not available. 908 participants were included in the analysis.

Interventions

Home oximetry monitoring was provided to participants with a known or suspected diagnosis of Covid-19. Participants were enrolled following attendance to accident and emergency departments, hospital admission or referral through primary care services.

Results

Of 908 patients enrolled into four different Covid-19 home oximetry programmes in England, 771 (84.9%) had oxygen saturations at rest of 95% or more, and 320 (35.2%) were under 65 years of age and without comorbidities. 52 (5.7%) presented to hospital and 28 (3.1%) died following enrolment, of which 14 (50%) had Covid-19 as a named cause of death. All-cause mortality was significantly higher in patients enrolled after admission to hospital (OR 8.70 [2.53 - 29.89]), compared to those enrolled in primary care. Patients enrolled after hospital discharge (OR 0.31 [0.15 - 0.68]) or emergency department presentation (OR 0.42 [0.20 - 0.89]) were significantly less likely to present to hospital than those enrolled in primary care.

Conclusions

This study find that home oximetry monitoring can be a safe pathway for Covid-19 patients; and indicates increases in risk to vulnerable groups and patients with oxygen saturations < 95% at enrolment, and in those enrolled on discharge from hospital. Findings from this evaluation have contributed to the national implementation of home oximetry across England.

For peer review only - http://bmjopen.bmj.com/site/about/guidelines.xhtml

Strengths and limitations of this study

- This multisite study examines outcomes and variation between four Covid-19 home oximetry remote monitoring pathways in England.
- It uses linked data from primary and secondary care, alongside mortality data and data collected from the oximetry pathway to understand clinical outcomes and prior risk factors.
- It examines the clinical status of patients enrolled under different home oximetry pathways and examines their rates of hospital attendance and all-cause mortality.
- The study is limited to four sites, and is unable to comment on the outcomes of patients not enrolled into these pathways or on the performance of pathways not included in the study.

For peer review only

Introduction

Before the outbreak of the Covid-19 pandemic, compelling evidence already existed regarding the role of telemedicine and digital technologies in restructuring how healthcare is delivered, indicating an opportunity to expand the use of virtual pathways [1]. Such evidence suggests that care delivered remotely can, in many circumstances, safely meet patients' clinical needs and personal preferences [1–3]. Specifically, remote monitoring pathways, those that rely on an initial point of contact with the health services followed by continuous monitoring via phone calls, digital or app-based diaries or wearable sensors, have also demonstrated effectiveness, especially when supported by behavioural change models [4–6]. However, the clinical effectiveness, safety and economic utility of this type of monitoring is context-dependent and varies considerably across clinical conditions; therefore, more evidence is required to fully assess their impact [4].

During the Covid-19 pandemic, the UK National Health Service (NHS), and health systems across the world, rapidly adopted novel remote monitoring pathways, many relying on home pulse oximetry [7–9]. These pathways were often initiated quickly in an attempt to proactively manage patients without risking unnecessary patient travel to hospital, but subsequently varied in terms of the devices used, patients included and the method of implementation. The available literature suggests potential for home management of Covid to support a positive patient experience [10–12]. However, while pulse oximetry and trends over time appear to be an effective way of detecting deterioration, the evidence surrounding the safety of oximetry devices, specifically low-cost pocket oximeters, is variable and more research is required to understand what oxygen saturation thresholds should trigger a patient to seek in-person care [13,14]. Overall, the literature surrounding whether home oximetry monitoring for Covid-19 patients is safe remains inconclusive [9,15].

In the UK, NHS England/Improvement, the body responsible for improved delivery of NHS care, in partnership with NHS Digital and Imperial College London, set out to understand, quantitatively, whether home oximetry monitoring was a safe clinical pathway to implement nationally. Following the first UK peak of Covid-19, in Spring 2020, pilots of home oximetry monitoring tested a system-wide approach to the early detection of Covid-19 in the community. As part of this pilot, a rapid evaluation was conducted to determine whether home oximetry monitoring was a safe clinical pathway. Based on existing literature, the initial hypothesis was that early recognition, escalation, admission and treatment could save lives in Covid-19 and would be a safe approach to adopt. In December 2020, informed by the findings of this study, NHS England and NHS Improvement recommended that all Clinical Commissioning Groups (CCGs) put in place a Covid-19 home oximetry service. At the time of writing, more than twenty thousand patients in England have been treated on similar pathways to those included in this study [16].

Aims & Objectives

This study aimed to answer the research question: *Are home oximetry monitoring pathways safe and effective for Covid-19 patients in the English NHS?*

The primary objective was to determine whether patients suffered any adverse consequences as a result of home oximetry monitoring. The secondary objective was to explore whether the recommendations relating to oxygen saturation that were published in June 2020 in the NHS Covid-19 assessment pathway were correct. The final objective was to contribute to a

For peer review only - http://bmjopen.bmj.com/site/about/guidelines.xhtml

recommendation to NHS England/Improvement regarding the safety and suitability of home oximetry monitoring as a national Covid-19 pathway.

Methods

Setting & Design

To answer this question, Imperial College NHS Healthcare Trust, NIHR Imperial Patient Safety Translational Research Centre (PSTRC) at the Institute of Global Health Innovation (IGHI) at Imperial College London, working in partnership with NHS Digital, assessed existing evidence concerning home oximetry monitoring for respiratory conditions and analysed prospective data collected from four sites.

This was a retrospective, multi-site, observational study of home oximetry monitoring for patients with suspected or proven Covid-19 in England during Summer 2020, including an analysis of patient data from four pilot sites: North West London, Slough, South Tees and Watford. Sites varied in how patients were enrolled onto pathways; from primary care, after a presentation to the emergency department or following a hospital admission, or a combination of these. In each site, patients were enrolled onto the oximetry pathway and were provided with a pulse oximeter to measure their oxygen saturations and heart rate over time. These recordings, in addition to a patient's symptoms and perceived overall wellbeing were communicated to their nominated healthcare professional (either a general practitioner, practice nurse or hospital doctor) through a combination of app-based platforms and paper diaries. The frequency of recording varied between pathways, from daily to several times per day. In the case of clinical deterioration or other concerns, patients were able to contact their nominated healthcare professional for advice and escalation of care could be initiated as appropriate. While services may vary between sites, the current Standard Operating Procedure for Covid-19 home oximetry services in England is included in **Supplement 1**.

Quantitative analysis was conducted in the IGHI's Big Data and Analytical Unit (BDAU). The BDAU provide a fully certified ISO 27001:2013 research environment within Imperial College and is fully compliant with NHS IG Toolkit Level 3 (EE133887). The work was conducted as a service evaluation, as institutional Research Governance deemed that it did not require further ethics committee approval. Information Governance approval was obtained and a data sharing agreement established (DARS-NIC-396113-N9L4L-v1.2). Analysis took place within the Imperial College London Big Data and Analytical Unit.

Information & Data

Four separate datasets linked by a pseudonymised patient identifier were provided by NHS Digital for the express purpose of this evaluation. A full list of variables collected is included in **Supplement 2** and summarised below:

Primary Care Records: Data pertaining to patient demographics and clinical comorbidities were obtained from the General Practice Extraction Service (GPES) Data for Pandemic Planning and Research (GDPPR) for all patients enrolled into a home oximetry monitoring programme at one of the four sites. Records were available from the start of a patient's primary care record in the practice to the date of data transfer (25th Sept 2020).

For peer review only - http://bmjopen.bmj.com/site/about/guidelines.xhtml

Home oximetry Monitoring Records: For each patient enrolled into a home oximetry monitoring programme at the four sites, data were collected from individual home oximetry monitoring providers consisting of the date of enrolment, oxygen saturations at rest at enrolment and the clinical pathway to which they were enrolled.

Hospital records: The dates, outcomes of Accident and Emergency presentations and admissions to hospital for patients while enrolled in the home oximetry programme were obtained from case note review by participating sites. Data were returned by sites in mid-September 2020.

Mortality records: The date and causes (as ICD-10 codes) of death were provided for each patient whose death had been recorded by the Office for National Statistics after enrolment to the home oximetry programme until the date of data transfer (25th Sept 2020).

Analysis & Statistical Procedure

Data were linked according to the pseudonymised patient identifier. A single cohort of patients was identified where GDPPR data and oxygen saturations at rest at enrolment were available. The demographic and clinical characteristics of this population where described and differences in patient and clinical characteristics were compared between routes of enrolment using pairwise Fisher's Exact Tests for count data and Mann Whitney U-tests for non-parametric continuous data.

The likelihood of a patient presenting to hospital at least once following enrolment to the home oximetry monitoring programme was examined using univariable and multivariable logistic regression models. The likelihood of all-cause mortality following enrolment to the home oximetry monitoring programme was also examined using univariable and multivariable logistic regression models. Frequent absence of dates of enrolment or dates of hospital admission precluded formal temporal evaluation of time to hospital presentation or mortality.

Results

A total of 1338 patients were recorded as being enrolled into the home oximetry programme at one of the four pilot sites. Of these, GDPPR records were available for 1242 patients (92.8%). Of these patients, a recorded oxygen saturation level at enrolment onto the home oximetry was present for 908 patients (73.1%).

Comparison of Enrolment Pathways

The characteristics of the included population, overall and according to the route of enrolment, are described in **Table 1**. 302 patients were enrolled from primary care (33.4%), 342 from A&E (37.9%) and 259 following discharge from hospital (28.9%). Route of enrolment was missing for five patients (0.6%).

Oxygen saturations at enrolment were significantly lower in those enrolled on discharge from hospital (96%) than through A&E (97%) or primary care (98%) (MWU, $p < 0.0001$). No difference was observed between primary care and A&E pathways (MWU, $p = 0.0852$). Similarly, there was no significant difference in the proportion of patients enrolled with oxygen

For peer review only - http://bmjopen.bmj.com/site/about/guidelines.xhtml

saturations of 95% or more in primary care (92.0%) or A&E (91.8%) (Fishers Exact Test, p = 1), however only 67.6% of patients enrolled after hospital discharge had oxygen saturations of 95% or more (pairwise Fishers Exact Tests, $p < 0.0001$).

Patients enrolled through A&E were most likely to have no comorbidities (47.1%) compared to primary care (37.4%) and hospital discharge (27.0). Primary care patients were less likely to have comorbidities than those enrolled after hospital discharge (pairwise Fishers Exact Tests, $p < 0.05$ in all cases). Patients enrolled through A&E were younger (median age = 50 years) than those enrolled in primary care (55 years) and on hospital discharge (63 years) (MWU, $p < 0.0001$ in all cases).

Presentation to Hospital

A total of 69 presentations to hospital were made by 52 patients (5.7%) after enrolment. 40 of these patients (76.9%) were admitted to hospital, and 8 patients (15.4%) presented more than once. The proportion of patients presenting to hospital according to age group and number of clinical comorbidities is shown in **Figure 1** and **Figure 2**.

3.1% of patients under the age of 65 years and without comorbidities presented to hospital, compared to 5% of those under 65 with comorbidities and 9.7% of those aged 65 and over. Similarly, 5.3% of patients with oxygen saturations of 95% or more at enrolment presented to hospital, compared to 6.1% of those with oxygen saturations of 93-94% and 10.9% of those with oxygen saturations less than 93% at enrolment (**Table 2**).

In univariable logistic regression models, presentation to hospital was significantly more likely in older patients and those with more clinical comorbidities, while those initiated on the home oximetry through A&E or on discharge from hospital were less likely to re-present to hospital (**Table 3**). In the multivariable model, increasing age (OR 1.03, p = 0.018) was associated with significantly higher odds of presentation to hospital, while those initiated through A&E (OR 0.42, p = 0.024) and following discharge from hospital (OR 0.31, p = 0.003) were significantly less likely to present to hospital (**Table 3**).

All-cause mortality

A total of 28 patients (3.1%) died of any cause following enrolment. 14 of these patients (50%) had Covid-19 as a named cause of death (ICD-10 code U07.1), and 12 (42.9%) had Covid-19 as the underlying cause of death. All-cause mortality by age group and number of clinical comorbidities is shown in **Figure 1** and **Figure 2**. Only patients with one or more comorbidities died, while 17 (60.1%) patients who died had four or more clinical comorbidities.

None of the 320 patients under the age of 65 and without comorbidities included in the study died during the study period, compared to 0.9% of those under 65 with comorbidities and 9.3% of those aged 65 and over. 2.6% of patients with oxygen saturations of 95% or more at enrolment died during the study period, compared to 6.1% of those with oxygen saturations of 93-94% and 5.5% of those with oxygen saturations less than 93% at enrolment (**Table 2**).

For peer review only - http://bmjopen.bmj.com/site/about/guidelines.xhtml

In univariable logistic regression models, all-cause mortality was significantly more likely in older patients, those of BAME ethnicity, with more clinical comorbidities, who were overweight or obese and those initiated on the home oximetry following discharge from hospital (**Table 4**). In the multivariable model, increasing age (OR 1.08, p = 0.000), more clinical comorbidities (OR 1.45, p = 0.009), being overweight or obese (OR 4.83, p = 0.002) and being initiated on the home oximetry following discharge from hospital (OR 8.70, p = 0.001) were associated with significantly higher odds of all-cause mortality (**Table 4**).

Discussion

Statement of key findings

In terms of clinical safety, only 5.7% of patients presented to hospital after enrolment. The likelihood of presenting to hospital increased with age, but was not significantly associated with ethnicity, number of clinical comorbidities, obesity or oxygen saturations at enrolment. However, all-cause mortality was significantly more likely with increasing age, number of clinical comorbidities and obesity. All-cause mortality was also higher for those patients who initiated the pathway after discharge from hospital. Finally, most patients using home oximetry monitoring were of low clinical severity on initiation and neither died nor presented to hospital during the study period. Therefore, the Covid-19 assessment pathway published in June 2020 is clinically appropriate, and its recommendations regarding oxygen saturation thresholds are correct.

While hospital presentation and mortality were associated with lower oxygen saturations at enrolment, this was not a statistically significant relationship after controlling for other patient factors. Therefore, although oxygen saturation at enrolment may provide an important tool by which to stratify clinical severity, such assessments must also incorporate a wider understanding of factors determining outcome including age, obesity and clinical comorbidities.

None of the 320 participants aged less than 65 and without comorbidities died during the study period. These individuals accounted for 33.3% of all participants which suggests that during the pilots a large proportion of the participants were low risk at enrolment and did not suffer any adverse outcome. Wider implementation should therefore focus on higher risk groups to ensure efficient use of limited health and care resources.

Statistically significant variation in the characteristics of patients, clinical severity of illness, rates of hospital presentation and all-cause mortality were observed between the three routes of enrolment. Patients enrolled following a hospital admission had 8.7 times higher odds of all cause mortality than patients enrolled in primary care after adjusting for age, sex, ethnicity, obesity, clinical comorbidities and oxygen saturation at enrolment. Conversely, compared to those enrolled in primary care, patients enrolled after an A&E presentation or hospital admission had significantly lower odds of further presentation to hospital after enrolment. Collectively, this indicates differences in patient characteristics and the risk of adverse events according to the route of enrolment. Those enrolled in primary care are generally the least unwell and go on to have lower rates of hospital presentation and mortality. The finding that

For peer review only - http://bmjopen.bmj.com/site/about/guidelines.xhtml

patients enrolled in A&E or following hospital admission have lower rates of hospital presentation may indicate reluctance on the part of patients to return to hospital during the same period of illness. Patients enrolled following hospital discharge appear to be at particularly high risk of mortality and may therefore represent a patient population that should either be offered more intensive monitoring or alternative approaches to reducing the risk of subsequent mortality.

Following enrolment more patients aged 90 years and over died during the study period than presented to hospital, while in younger age groups hospital presentations outnumbered all-cause mortality (Figure 1). A similar trend was observed according to increasing clinical comorbidities (Figure 2). This is likely to reflect circumstances in which hospital admission is not in accordance with a patient's wishes or considered clinically appropriate, and end-of-life care is therefore initiated in a person's usual place of residence.

Taken together, our results support the hypothesis that oximetry monitoring is a safe pathway for Covid-19 patients. This was reported to NHS England and Improvement in October 2020.

Strengths & Limitations

The primary strength of this study is its system-level and patient-level impact, since this work evaluated a real-world pilot of a new clinical pathway. The project team achieved an integrated partnership between academics, clinicians and policy makers. This enabled a direct pipeline from evidence generation to policy decision making; moreover, the efficiency with which the work was conducted was nationally important, as it was required to inform how the NHS would use home oximetry monitoring during the next wave of the pandemic. Impact was achieved through the unique composition of the evaluation team as well as the close relationship the team held to NHS leaders in home oximetry monitoring. The method for this work required novel data reporting from the pilot sites to the evaluation team and this was ensured through close collaboration with the individual sites.

The most prevalent limitation related to data quality and completeness. The pilot sites varied in their routes of referral and in the clinical severity of the patients they enrolled. Furthermore, there was heterogeneity in the actual intervention across the sites, as some enrolled patients with suspected Covid-19, while others included patients who had received hospital treatment for Covid-19 and were subsequently discharged. Due to the rapid timescales of this evaluation and the pressured environment in which it took place, mandating full completeness in the data submitted was not possible, and the resulting dataset had a considerable amount of missing data. The lack of longitudinal oxygen saturations data precluded temporal analysis of patient trajectories and identification of deterioration in clinical status. While such an evaluation would have been valuable, it was not possible within the available dataset.

In addition, the absence of precise temporal recording of data and an inability to complete individual case note reviews precluded a more detailed evaluation of whether deaths occurred as a result of Covid-19 infection, or another cause entirely. Finally, this was not a controlled study, which prevented a comparative analysis. Due to these limitations and the relatively low community prevalence of Covid-19 during the pilot, our findings are not necessarily generalisable to future waves of Covid-19. Additionally, while many initial oxygen saturations

For peer review only - http://bmjopen.bmj.com/site/about/guidelines.xhtml

are likely to be taken in the presence of a healthcare professional, we are unable to determine whether these readings are taken correctly and may therefore provide inappropriate estimates of clinical acuity.

Comparison to other studies

While research evidence regarding Covid-19 home oximetry is still in its infancy, this study reflects similar findings in the published literature. One systematic review of home monitoring for Covid-19 acknowledges inconclusive evidence about effectiveness and safety. While this study could not conclusively determine the clinical effectiveness of the pilots, it was able to demonstrate non-inferiority in terms of safety with traditional hospital management of Covid-19.

Furthermore, this study indicates the potential for these technologies to support pandemic management in line with expert opinions published in the literature [17]. The findings of this work are consistent with existing grey literature suggesting the utility of home oximetry monitoring for Covid-19 [8,18]. This study specifically assessed the safety of home oximetry for Covid-19 and makes policy recommendations not present in existing studies which tend to focus more on patient experience and potential clinical effectiveness. These findings echo the findings of a previous systematic review indicating a paucity of studies directly addressing patient safety in telemedicine [19]. Identifying the potential patient safety risks arising from the national home oximetry programme is an area of ongoing qualitative and quantitative research as part of the national Covid-19 Oximetry at Home Programme [18].

Implications for Policy

This work was explicitly designed to answer relevant questions in advance of the national implementation of a new clinical pathway for Covid-19. It confirms the appropriateness of existing policies and that safety netting approaches are congruent with findings.

This study provides information as to how the pathway should function including the thresholds at which people should be enrolled into home oximetry monitoring programmes, how long they should continue in home oximetry monitoring and what sorts of technology is required to support home oximetry monitoring for Covid-19.

Furthermore, the practicalities of running this study revealed important considerations for the ongoing evaluation of home oximetry monitoring pathways for Covid-19. It is clear from the study that it is only possible to measure the safety of home oximetry monitoring with the robust and rigorous collection of data from healthcare providers as well as any third party app-based providers. This data flow should continue during national implementation to continuously measure safety under different conditions and levels of organisational pressure.

Conclusion

Advancing the evidence base regarding the safety of home oximetry monitoring for Covid-19 patients is of considerable importance as many health systems face new waves of the pandemic worldwide. This study reveals, via a real-world pilot evaluation, that home oximetry monitoring can be a safe pathway for Covid patients; however, substantial research is needed

For peer review only

to understand its clinical effectiveness across patient populations. This study was limited by complex and incomplete data as well as variation in intervention designs; a product of the pandemic context within which it was undertaken. However, it did provide initial evidence of the appropriate clinical thresholds and patient characteristics for home oximetry. It also indicated increases in risk to vulnerable groups and patients with oxygen saturations below 95% at enrolment.

Findings from this evaluation have contributed to the national implementation of home oximetry across England, and further work will be undertaken to evaluate clinical effectiveness and any inequalities in terms of access to, and inclusion in, the new pathway.

For peer review only

For peer review only - http://bmjopen.bmj.com/site/about/guidelines.xhtml

Funding

This research was supported by the National Institute for Health Research (NIHR) Imperial Patient Safety Translational Research Centre (PSTRC). The views expressed are those of the authors and not necessarily those of the NHS, the NIHR or the Department of Health and Social Care. JC acknowledges support from the Wellcome Trust (215938/Z/19/Z).

Conflicts of Interest

All authors have completed the ICMJE uniform disclosure form at www.icmje.org/coi_disclosure.pdf and have no competing interests to declare.

Acknowledgements

The research team would like to thank the teams at NHS Digital and NHS England/Improvement as well as the site leads from the home oximetry pilot sites.

Data Sharing Statement

Patient-level pseudonymised data were obtained from NHS Digital for the specific purpose of the evaluation. Aggregate findings are presented in this study. Patient-level data may be obtained through application and review by NHS Digital.

Contribution Statement

JC, KF and RC were involved in all aspects of the study. HA, GF, JB, AD and SE were involved in the conceptualisation of the study, interpretation of findings and in the reviewing and editing of the draft. JC has had access to all the data in the study and all authors had final responsibility for the decision to submit for publication. JC attests that all listed authors meet authorship criteria and that no others meeting the criteria have been omitted.

Copyright Statement

The Corresponding Author has the right to grant on behalf of all authors and does grant on behalf of all authors, a worldwide licence to the Publishers and its licensees in perpetuity, in all forms, formats and media (whether known now or created in the future), to i) publish, reproduce, distribute, display and store the Contribution, ii) translate the Contribution into other languages, create adaptations, reprints, include within collections and create summaries, extracts and/or, abstracts of the Contribution, iii) create any other derivative work(s) based on the Contribution, iv) to exploit all subsidiary rights in the Contribution, v) the inclusion of electronic links from the Contribution to third party material where-ever it may be located; and, vi) licence any third party to do any or all of the above.

Patient and Public Involvement Statement

Patients and/or the public were not involved in the design, or conduct, or reporting, or dissemination plans of this research.

For peer review only

Transparency Declaration

JC affirms that the manuscript is an honest, accurate, and transparent account of the study being reported; that no important aspects of the study have been omitted; and that any discrepancies from the study as originally planned (and, if relevant, registered) have been explained.

Ethics Approval Statement

The work was conducted as a service evaluation, as institutional the Imperial College Research Ethics Committee deemed that it did not require further ethics committee approval.

For peer review only

For peer review only - http://bmjopen.bmj.com/site/about/guidelines.xhtml

Citations

1 Greenhalgh T, Vijayaraghavan S, Wherton J, *et al.* Virtual online consultations: Advantages and limitations (VOCAL) study. *BMJ Open* 2016;**6**:e009388. doi:10.1136/bmjopen-2015-009388

2 Personalised Health and Care 2020 Using Data and Technology to Transform Outcomes for Patients and Citizens A Framework for Action. 2014.

3 Joshi M, Ashrafian H, Arora S, *et al.* Digital alerting and outcomes in patients with sepsis: Systematic review and meta-analysis. J. Med. Internet Res. 2019;**21**:e15166. doi:10.2196/15166

4 Noah B, Keller MS, Mosadeghi S, *et al.* Impact of remote patient monitoring on clinical outcomes: an updated meta-analysis of randomized controlled trials. npj Digit. Med. 2018;**1**:1–12. doi:10.1038/s41746-017-0002-4

5 Kim YN, Shin DG, Park S, *et al.* Randomized clinical trial to assess the effectiveness of remote patient monitoring and physician care in reducing office blood pressure. *Hypertens Res* 2015;**38**:491–7. doi:10.1038/hr.2015.32

6 Iqbal FM, Joshi M, Davies G, *et al.* Design of the pilot, proof of concept REMOTE-COVID trial: remote monitoring use in suspected cases of COVID-19 (SARS-CoV-2). *Pilot Feasibility Stud* 2021;**7**:1–7. doi:10.1186/s40814-021-00804-4

7 Medina M, Babiuch C, Card M, *et al.* Home monitoring for COVID-19: Posted April 18, 2020. *Cleve Clin J Med* 2020;**87**:1–4. doi:10.3949/CCJM.87A.CCC028

8 Coronavirus » Pulse oximetry to detect early deterioration of patients with COVID-19 in primary and community care settings. https://www.england.nhs.uk/coronavirus/publication/pulse-oximetry-to-detect-early-deterioration-of-patients-with-covid-19-in-primary-and-community-care-settings/ (accessed 12 May 2021).

9 Gerke S, Shachar C, Chai PR, *et al.* Regulatory, safety, and privacy concerns of home monitoring technologies during COVID-19. *Nat Med* 2020;**26**:1176–82. doi:10.1038/s41591-020-0994-1

10 Morgan AU, Balachandran M, Do D, *et al.* Remote Monitoring of Patients with Covid-19: Design, implementation, and outcomes of the first 3,000 patients in COVID Watch. *Nejm Catal Innov Care Deliv* doi:10.1056/CAT.20.0342

11 Taiwo O, Ezugwu AE. Smart healthcare support for remote patient monitoring during covid-19 quarantine. *Informatics Med Unlocked* 2020;**20**:100428. doi:10.1016/j.imu.2020.100428

12 Watson AR, Wah R, Thamman R. The value of remote monitoring for the COVID-19 pandemic. *Telemed e-Health* 2020;**26**:1110–2. doi:10.1089/tmj.2020.0134

13 Levitan RM. Pulse Oximetry as a Biomarker for Early Identification and Hospitalization of COVID-19 Pneumonia. Acad. Emerg. Med. 2020;**27**:785–6. doi:10.1111/acem.14052

14 Luks AM, Swenson ER. Pulse oximetry for monitoring patients with COVID-19 at home potential pitfalls and practical guidance. Ann. Am. Thorac. Soc. 2020;**17**:1040–6. doi:10.1513/AnnalsATS.202005-418FR

15 Vindrola-Padros C, Singh KE, Sidhu MS, *et al.* Remote home monitoring (virtual wards) during the COVID-19 pandemic: A systematic review. medRxiv. 2020;:2020.10.07.20208587. doi:10.1101/2020.10.07.20208587

For peer review only

16 COVID Oximetry @home - Management Information File 6th May 2021 - NHS Digital. https://digital.nhs.uk/data-and-information/supplementary-information/2021/covid-oximetry-at-home---management-information-file-6-may-2021 (accessed 18 May 2021).

17 Zhao M, Wasfy JH, Singh JP. Sensor-aided continuous care and self-management: implications for the post-COVID era. Lancet Digit. Heal. 2020;**2**:e632–4. doi:10.1016/S2589-7500(20)30220-X

18 COVID Oximetry @home - digital and data services - NHS Digital. https://digital.nhs.uk/coronavirus/covid-oximetry-at-home-digital-and-data-services (accessed 12 May 2021).

19 Guise V, Anderson J, Wiig S. Patient safety risks associated with telecare: A systematic review and narrative synthesis of the literature. *BMC Health Serv Res* 2014;**14**:1–15. doi:10.1186/s12913-014-0588-z

For peer review only

Figures:

Figure 1: Percentage all-cause mortality and presentation to hospital according to patient age

Figure 2: Percentage all-cause mortality and presentation to hospital according to number of clinical comorbidities

For peer review only

Tables:

		Primary Care	Accident & Emergency	Inpatient discharge	Overall
Total Patients		302	342	259	908
Female		180 (59.6)	205 (59.9)	120 (46.3)	508 (55.9)
Median Age (years)		55	50	63	54
Ethnicity	White	113 (37.4)	160 (46.8)	140 (54.1)	415 (45.7)
	Black, Asian or Minority Ethnic	82 (27.2)	103 (30.1)	59 (22.8)	244 (26.9)
	Not recorded	107 (35.4)	79 (23.1)	60 (23.2)	249 (27.4)
Overweight or obese		108 (35.8)	189 (55.3)	160 (61.8)	458 (50.4)
Median oxygen saturations		98	97	96	97
Clinical severity based on oxygen saturations	Mild (≥ 95%)	278 (92.1)	314 (91.8)	175 (67.6)	771 (84.9)
	Moderate (93-94%)	17 (5.6)	22 (6.4)	42 (16.2)	82 (9.0)
	Severe (≤ 92%)	7 (2.3)	6 (1.8)	42 (16.2)	55 (6.1)
Number of clinical comorbidities	0	113 (37.4)	161 (47.1)	70 (27.0)	346 (38.1)
	1	75 (24.8)	105 (30.7)	70 (27.0)	252 (27.7)
	2	42 (13.9)	45 (13.2)	44 (17.0)	131 (14.4)
	3+	72 (23.8)	31 (9.1)	75 (29.0)	179 (19.7)
Hypertension		102 (33.8)	65 (19.0)	111 (42.9)	278 (30.8)
Depression		86 (28.5)	84 (24.6)	72 (27.8)	242 (26.8)
Asthma		75 (24.8)	83 (24.3)	49 (18.9)	207 (22.9)
Steroid use		87 (28.8)	62 (18.1)	54 (20.8)	203 (22.5)
Diabetes mellitus		56 (18.5)	43 (12.6)	63 (24.3)	162 (17.9)
Pregnancy		37 (12.3)	74 (21.6)	23 (8.9)	134 (14.8)
Chronic heart disease		43 (14.2)	35 (10.2)	55 (21.2)	133 (14.7)
Mild frailty		41 (13.6)	17 (5.0)	42 (16.2)	100 (11.1)
Moderate frailty		45 (14.9)	8 (2.3)	43 (16.6)	96 (10.6)
Chronic respiratory disease		39 (12.9)	17 (5.0)	35 (13.5)	91 (10.1)
Chronic kidney disease		32 (10.6)	17 (5.0)	41 (15.8)	90 (10.0)
Cancer		32 (10.6)	23 (6.7)	23 (8.9)	78 (8.6)
Hypothyroidism		29 (9.6)	22 (6.4)	17 (6.6)	68 (7.5)
Hypercholesterolemia		30 (9.9)	14 (4.1)	22 (8.5)	66 (7.3)
Chronic neurological disease		28 (9.3)	7 (2.0)	25 (9.7)	60 (6.6)
Severe frailty		*	*	*	53 (5.9)
Stroke		*	*	*	46 (5.1)
Immunosuppression drug		8 (2.6)	12 (3.5)	7 (2.7)	27 (3.0)
Dementia		*	*	*	25 (2.8)
Mental illness		8 (2.6)	6 (1.8)	8 (3.1)	22 (2.4)
Epilepsy		*	*	*	20 (2.2)
Peripheral vascular disease		*	*	*	13 (1.4)
Autoimmune disease		*	*	*	13 (1.4)
Chronic liver disease		*	*	*	9 (1.0)
Learning disability		*	*	*	6 (0.7)

For peer review only

Table 1: Characteristics of the study population, overall and according to the route of enrolment. NB: Route of enrolment was missing for 5 of 908 patients. Comorbidity case numbers are not presented for routes of enrolment where one or more route of enrolment involved 5 of fewer patients.

For peer review only

Table 2: Number of patients, frequency of hospital presentation and all-cause mortality according to risk groups and oxygen saturations at enrolment

Outcome	Risk Group	Oxygen Saturations Severity			
		Mild (≥ 95%)	Moderate (93-94%)	Severe (≤ 92%)	Overall
Total Patients	>= 65 years	203 (75.5)	37 (13.8)	29 (10.8)	269 (29.6)
	< 65 years with comorbidities	277 (86.8)	29 (9.1)	13 (4.1)	319 (35.1)
	< 65 years without comorbidities	291 (90.9)	16 (5.0)	13 (4.1)	320 (35.2)
	Overall	771 (84.9)	82 (9.0)	55 (6.1)	908 (100.0)
Hospital presentation	>= 65 years	18 (8.9)	3 (8.1)	5 (17.2)	26 (9.7)
	< 65 years with comorbidities	13 (4.7)	2 (6.9)	1 (7.7)	16 (5.0)
	< 65 years without comorbidities	10 (3.4)	0 (0.0)	0 (0.0)	10 (3.1)
	Overall	41 (5.3)	5 (6.1)	6 (10.9)	52 (5.7)
All-cause mortality	>= 65 years	18 (8.9)	4 (10.8)	3 (10.3)	25 (9.3)
	< 65 years with comorbidities	2 (0.7)	1 (3.4)	0 (0.0)	3 (0.9)
	< 65 years without comorbidities	0 (0.0)	0 (0.0)	0 (0.0)	0 (0.0)
	Overall	20 (2.6)	5 (6.1)	3 (5.5)	28 (3.1)

For peer review only

Table 3: Univariable and multivariable binary logistic regression output for presentation to hospital following enrolment to the home oximetry pathway

		Univariable			Multivariable		
		OR	p-value	95% CI of OR	OR	p-value	95% CI of OR
Age		1.03	**0.000**	1.01 - 1.05	1.03	**0.018**	1.00 - 1.05
Ethnicity	White	reference			reference		
	Black, Asian and Minority Ethnic	1.22	0.544	0.64 - 2.32	1.57	0.198	0.79 - 3.10
	Not recorded	0.75	0.447	0.36 - 1.57	0.95	0.912	0.42 - 2.17
Number of comorbidities		1.25	**0.004**	1.08 - 1.45	1.07	0.503	0.88 - 1.30
Overweight or Obese		0.67	0.175	0.38 - 1.19	0.55	0.069	0.29 - 1.05
Clinical severity based on oxygen saturations	Mild (≥ 95%)	reference			reference		
	Moderate (93-94%)	1.16	0.766	0.44 - 3.01	1.08	0.885	0.40 - 2.92
	Severe (≤ 92%)	2.18	0.091	0.88 - 5.39	2.35	0.096	0.86 - 6.46
Enrolment Pathway	Primary care	reference			reference		
	Accident & Emergency	0.37	**0.005**	0.18 - 0.74	0.42	**0.024**	0.20 - 0.89
	Inpatient	0.49	**0.049**	0.25 - 0.98	0.31	**0.003**	0.15 - 0.68

For peer review only - http://bmjopen.bmj.com/site/about/guidelines.xhtml

Table 4: Univariable and multivariable binary logistic regression output for all-cause mortality following enrolment to the home oximetry pathway

		Univariable			Multivariable		
		OR	p	95% CI of OR	OR	p	95% CI of OR
Age		1.12	**0.000**	1.08 - 1.16	1.08	**0.000**	1.03 - 1.13
Ethnicity	White	reference			reference		
	Black, Asian and Minority Ethnic	0.16	**0.015**	0.04 - 0.70	0.37	0.235	0.07 - 1.90
	Not recorded	0.49	0.129	0.19 - 1.23	0.61	0.371	0.21 - 1.79
Number of comorbidities		1.97	**0.000**	1.63 - 2.40	1.45	**0.009**	1.09 - 1.92
Overweight or Obese		3.87	**0.004**	1.56 - 9.64	4.83	**0.002**	1.74 - 13.37
Clinical severity based on oxygen saturations	Mild (≥ 95%)	reference			reference		
	Moderate (93-94%)	2.43	0.083	0.89 - 6.68	1.11	0.864	0.34 - 3.67
	Severe (≤ 92%)	2.16	0.224	0.62 - 7.53	0.65	0.574	0.14 - 2.93
Enrolment Pathway	Primary care	reference			reference		
	Accident &Emergency	0.66	0.587	0.15 - 2.97	3.40	0.157	0.62 - 18.55
	Inpatient	6.57	**0.001**	2.23 - 19.41	8.70	**0.001**	2.53 - 29.89

For peer review only

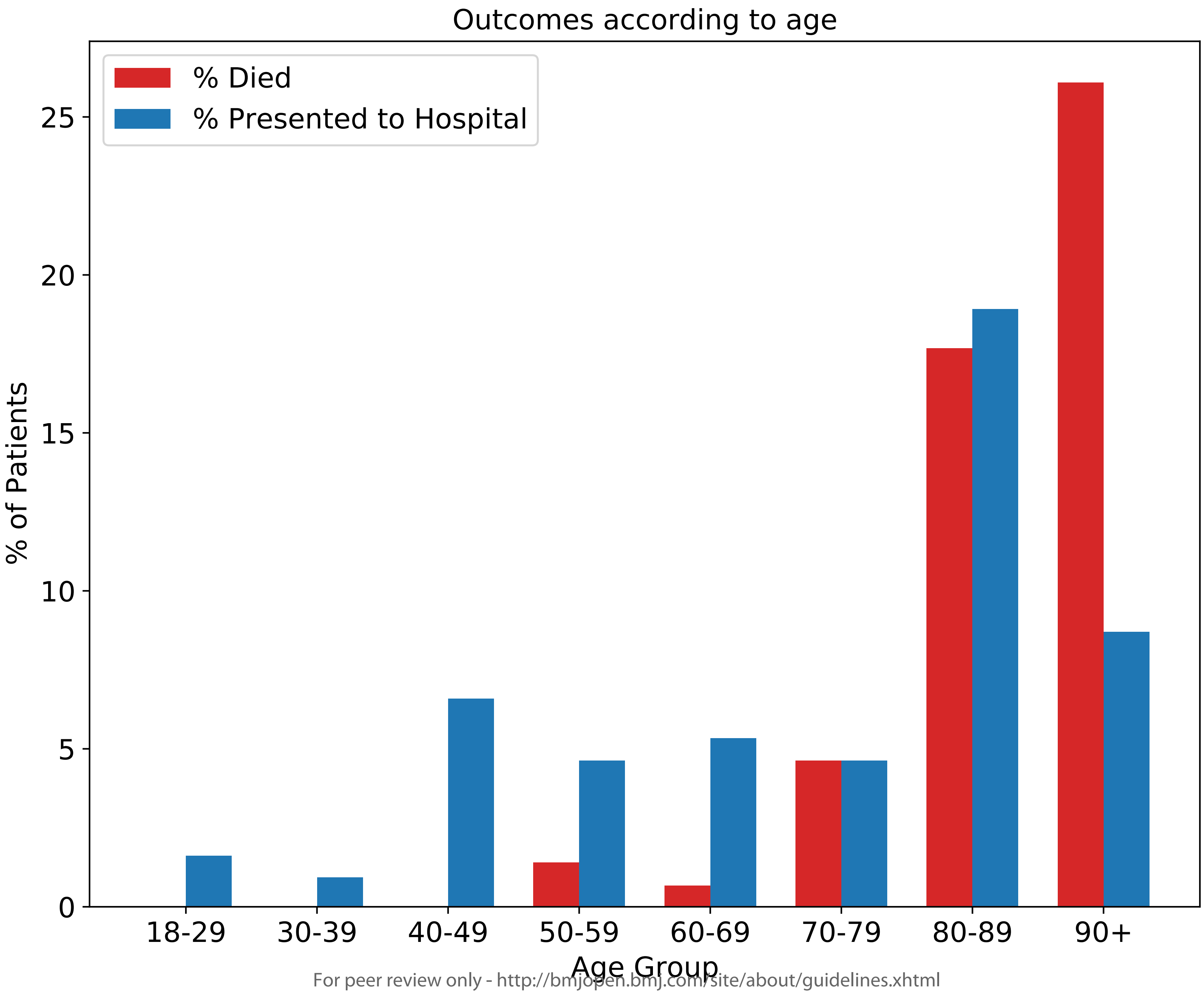
Outcomes according to age
% Died
% Presented to Hospital
% of Patients
0
5
10
15
20
25
18-29
30-39
40-49
50-59
60-69
70-79
80-89
90+
Age Group

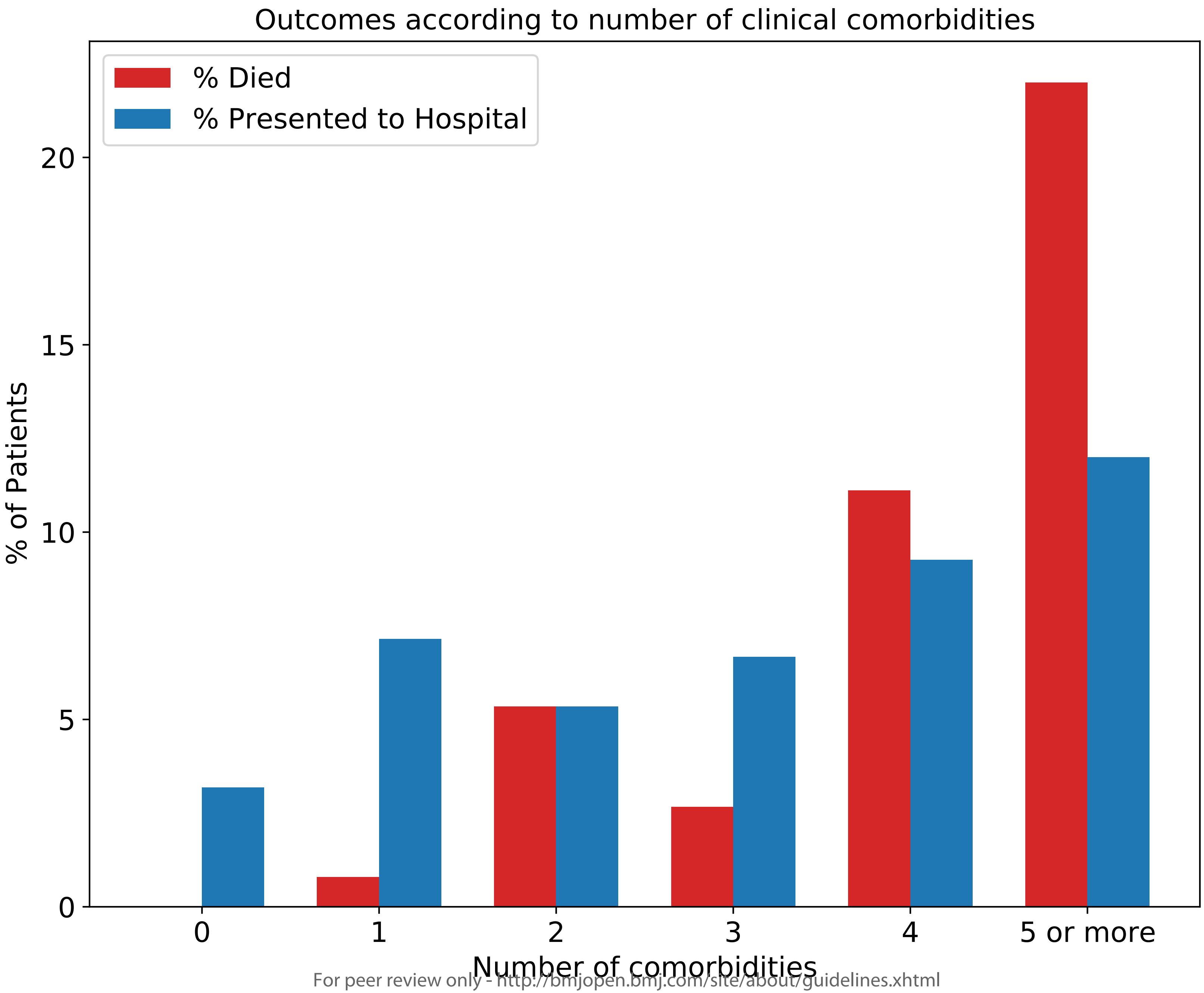
Outcomes according to number of clinical comorbidities
% Died
% Presented to Hospital
% of Patients
0
5
10
15
20
0
1
2
3
4
5 or more
Number of comorbidities

Classification: Official

Publications approval reference: 001559

Version 1.1,01 March 2021

Novel coronavirus (COVID-19) standard operating procedure

COVID Oximetry @home

This guidance is correct at the time of publishing. However, as it is subject to updates, please use the hyperlinks to confirm the information you are disseminating to the public is accurate.

Updates to the SOP published on 12 November 2020 are highlighted in yellow.

For peer review

1.1 Requirement

In November 2020, clinical commissioning groups (CCGs) were recommended to put in place a 'COVID Oximetry @home' model as rapidly as possible, further to guidance on the use of pulse oximetry first published in June 2020.

This document sets out a base standard for patient self-monitoring. It should not supplant existing arrangements where these are already established and working.

1.2 Entry criteria

The COVID Oximetry @home pathway should be available to people who are:

i. Diagnosed with COVID-19: either clinically or positive test result **AND**
ii. Symptomatic **AND EITHER**
iii. Aged 65 years or older **OR**
iv. Under 65 years and clinically extremely vulnerable (CEV) to COVID ~~(The Clinically Extremely Vulnerable to COVID list should be used as the primary guide. Clinical judgement can apply and take into account~~ or where clinical judgement applies, taking into account multiple additional COVID risk factors. ~~; for the most part, it is anticipated that this will already have led to inclusion on the CEV list~~. National criteria for inclusion on the CEV list are set and updated by government.

~~The national support offer, including oximeter supply, is targeted at this defined cohort.~~

1.3 Staffing and oversight

The default assumption is that the model is primarily implemented in general practice, eg including in hot hubs, working with community teams. NHS England and NHS Improvement have in parallel written to CCGs about supplementary funding arrangements to expand general practice capacity. (£150 million of additional funding has been allocated via CCGs to support GP capacity between now and March.) Referrals of the defined cohorts will also come via 111 COVID Clinical Assessment Service (CCAS), NHS Test and Trace and hospital emergency departments.

Legal responsibility, including ensuring appropriate clinical governance, remains with the relevant CCG. Each CCG should have a named person responsible for the establishment of the service in their area. Clinical, governance and administrative responsibilities included in the pathway can be provided by any appropriately trained person and best use of resources should be made, including asking NHS

For peer review only - http://bmjopen.bmj.com/site/about/guidelines.xhtml

Volunteer Responders to transport oximeters and patient packs, and the use of standard scripts to enable non-clinical staff (eg healthcare assistants, care navigators or volunteers) to undertake appropriate activities.

Where relevant, patients may benefit from a review of long-term condition management in the context of acute COVID illness. The responsibility for this lies with the patient's primary care physician and/or hospital specialist.

1.4 Patient journey

Referral (Stage 1)

- Systems should ensure timely referral of patients who may meet the entry requirements from all relevant providers operating within their area, eg NHS 111, Covid Clinical Assessment Service (CCAS), Test and Trace and hospital emergency departments.
- Arrangements will vary depending on how the pathway is delivered, eg through individual primary care networks or a single community health service.
- Patients should be advised to self-isolate in line with current guidance.

Triage (Stage 2)

- Patients referred to the service should have a standard assessment (with potential for face-to-face clinical assessment if deemed necessary), with shared decision-making prior to entry onto the pathway and a discussion about any support requirements for patients or carers. This should happen as soon as possible, and ideally on the same day as the referral.

  a. If at a hot site, then assessment should be done face to face and a baseline pulse oximetry reading taken; consider home oximetry monitoring if oxygen saturation levels are 95% or higher and proceed to stage 3. If oxygen levels are 94% or less, consider further clinical assessment, or proceeding to stage 3 with the option of more intense clinical assessment and oversight in the community, or hospital admission, eg if 92% or less.
  b. If contacted by phone or video, consider virtual assessment using a standard questionnaire and the need for a baseline oximetry reading either by visiting a hot site or home visit.

Onboarding (stage 3)

- Patients entering the pathway should be provided with a pulse oximeter and supporting information (including a paper diary which is ~~being made~~ available in

For peer review only - http://bmjopen.bmj.com/site/about/guidelines.xhtml

accessible formats and a variety of languages, or suitable app/regular call mechanism), contact details to report oximetry reading/symptoms, and clear safety netting instructions both in and out of hours. This should be supplied immediately if the patient is seen face to face or within 12 hours if the patient is assessed remotely. Patients should be instructed to attend their nearest emergency department within an hour, or call 999 if their saturation reading is 92% or less, or to contact 111/GP if 93% or 94%.

- For patients not seen face to face, NHS Volunteer Responders are available to help transport oximeters from a locally agreed location to the patient's home. Further details on this can be found at: https://nhsvolunteerresponders.org.uk/referral
- Patients should be encouraged to record oximetry readings daily, usually three times a day. Through a shared decision-making conversation, they are also given the option of a prompt at days 2, 5, 7, 10 and 12, either by (a) text message or (b) by e-mail, or instead (c) a non clinician-led check-in phone call.
- Patients should have clear instructions regarding the recognition of deterioration and instructions on the appropriate course of action, with 24/7 access to advice and support. Contact details must be communicated clearly to patients.
- Patients should agree in advance how they will return the oximeter, eg by either the practice or the patient arranging an NHS Volunteer Responder.

Monitoring (stage 4)

- Patients should receive text or email prompts, or check-in calls, as agreed during onboarding. A model message and phone script can be found on the NHS @home Future NHS platform (link below).
- Check-in calls should confirm that the patient is using the oximeter and diary correctly. If the phone call is clinician led, they may also be used to confirm the readings are 95% or above. The frequency of these calls can be reviewed with the patient if appropriate.

Recovery and discharge (stage 5)

- Patients who do not show signs of deterioration within 14 days of onset of symptoms should be actively discharged and supplied with leaving information, safety netting and safe advice on how to return the oximeter (eg to hot site, by a friend or family member, or through NHS Volunteer Responders). Model advice can be found on the NHS @home Future NHS platform (link below)
- Patients may be on the pathway for a shorter period either if they have been awaiting a test result and this is negative, or subject to clinical review.

For peer review only

For peer review only - http://bmjopen.bmj.com/site/about/guidelines.xhtml

- Patients who remain symptomatic at 14 days should receive a further clinical assessment and action taken as clinically appropriate.
- At the end of this stage, a friend or family member, or an NHS Volunteer Responder, collects and returns the oximeter for decontamination and reuse, as agreed as part of the onboarding conversation.

1.5 Oximeter supply and safe re-use

Oximeters for home use must meet ISO 80601-2-61:2017 and be CE marked.

Particular care needs to be given to ensuring reliable arrangements are in place for same-day oximeter distribution to patients, and their subsequent decontamination and reuse. Cleaning procedures for oximeters must follow manufacturers' instructions. Liquids should generally not be used on these devices due to the risk of fluid ingress damaging circuits. Disinfectant wipes should be used where possible.

A supply of pulse oximeters is available to CCGs based on national modelling assumptions of case demand.~~using the entry criteria in section 1.2 above. If local systems wish to extend the service beyond the defined groups, they will need to resource this locally, including provision of additional oximeters.~~
CCGs can request suitable oximeters from NHS Supply Chain in batches of 100, to be stored locally as appropriate for anticipated demand. Oximeters can be requested by emailing england.home@nhs.net. Once the order is agreed, delivery to the requesting CCG should be made within three working days. When requesting oximeters it is important to include the population numbers covered and where the service is sited.

Prior to being distributed to patients, and on return from them, oximeters must be decontaminated in line with infection control policies for reusable electronic equipment. They must be checked that they are functional and safe for re-use prior to being allocated to new patients. This should be done in line with local and national guidance for reusable electronic clinical monitoring equipment.

It is important to note that CCGs must ensure there are wholly reliable local arrangements in place for timely distribution, decontamination and re-use of sufficient oximeters.

Oximeters must be available for same-day distribution to patients, including out-of-hours. Patients should ideally not to have to wait more than 12 hours to receive an NHS oximeter.

For peer review only - http://bmjopen.bmj.com/site/about/guidelines.xhtml

1.6 Care homes

People living in care homes should receive the same standard of care as someone in their own home. This should be facilitated by care home staff and other supporting services. This should include full escalation or emergency admission or potential emergency home oxygen treatment and palliative treatments where appropriate.

Training and support for using pulse oximetry is available via the Care Provider Alliance and e-Learning for Healthcare resources. The COVID Oximetry @home monitoring diary has also been tailored for care home usage (see NHS @home Future NHS platform – link below). Any further support required in setting up the pathway within the care home can be provided through the care home's named clinical lead in the first instance.

1.7 Coding, record keeping and data requirements

SNOMED codes specific to home monitoring of COVID-19 patients can be accessed on the NHS Digital website. A number of templates have been created by local integrated care systems (ICS) as well as by Ardens, EMIS, TPP, Cegedim and accuRx.

All relevant information should be recorded in the patient record, including if a patient declines the pathway.

NHS Digital is sharing data with COVID Oximetry @home providers to assist in identifying patients who may be onboarded onto local pathways. To receive this daily list please contact dataliaison@nhs.net.

COVID Oximetry @home providers are required to submit data on COVID Oximetry @home patients on a weekly basis to NHS Digital SDCS. For further information or support please contact dataliaison@nhs.net.

1.8 Further support

Details of further advice, guidance and training materials including academic health science networks (AHSNs) and patient safety collaborative contacts for bespoke support is available on the NHS @home Future NHS platform: https://future.nhs.uk/NHSatH/grouphome.
Any safety concerns regarding oximeters should be reported to the MHRA via the COVID yellow card scheme (select 'other devices/equipment'; https://coronavirus-yellowcard.mhra.gov.uk/) and NHS England and NHS Improvement informed

For peer review only - http://bmjopen.bmj.com/site/about/guidelines.xhtml

(england.home@nhs.net). Local clinical engineering departments and medical device safety officers (MDSOs) should also be informed.

If there are any issues accessing the site or for further queries, please email england.home@nhs.net.

For peer review only

For peer review only - http://bmjopen.bmj.com/site/about/guidelines.xhtml

Category	Variable	Source	Data Type
Baseline Data	Age	PDS	Numeric
	Ethnicity	PDS	Categorical
	Sex	PDS	Categorical
	post code	PDS	String
	occupation	Onboarding	Categorical /string
	Registered GP Practice	PDS	String
	Home circumstances	Onboarding	Categorical
	Smoker	Onboarding / GP	Categorical
	**Clinical Comorbidities:***		
	BMI	Onboarding / GP	Numeric
	Asthma	Onboarding / GP	Binary
	COPD	Onboarding / GP	Binary
	Emphysema	Onboarding / GP	Binary
	Bronchiectasis	Onboarding / GP	Binary
	Other Chronic Resp Disease	Onboarding / GP	Binary / string
	Chronic cardiac disease	Onboarding / GP	Binary
	Chronic liver disease	Onboarding / GP	Binary
	Stroke or dementia	Onboarding / GP	Binary
	Neurological	Onboarding / GP	Binary
	Chronic kidney disease	Onboarding / GP	Binary
	Hypertension	Onboarding / GP	Binary
	Diabetes	Onboarding / GP	Binary
	Cancer	Onboarding / GP	Binary
	Cancer site(s) / types	Onboarding / GP	String
	Date of Cancer Diagnosis	Onboarding / GP	Date
	HIV	Onboarding / GP	Binary
	Transplant receipient	Onboarding / GP	Binary
	Transplant sites	Onboarding / GP	Cateogrical
	Immunosuppression regimen	Onboarding / GP	String
	**Onset of Symptoms:**		

Initial Entry	Breathlessness	Onboarding	Date
	Myalgia	Onboarding	Date
	Chills	Onboarding	Date
	Severe fatigue	Onboarding	Date
	Sputum	Onboarding	Date
	Dizziness	Onboarding	Date
	Cough	Onboarding	Date
	Nausea or vomiting	Onboarding	Date
	Diarrhoea	Onboarding	Date
	Headache	Onboarding	Date
	Sore throat	Onboarding	Date
	Nasal congestion	Onboarding	Date
	Anosmia	Onboarding	Date
	Unwell contact	Onboarding	Binary
	Sars COV 2 swab positive	Onboarding	Categorical
	Referral source	Onboarding	Categorical
	Date and time of recording	Onboarding	Timestamp
	**Clinical observations:**		
	O2 sats at rest	Onboarding	Numerical
	O2 sats after exertion	Onboarding	Numerical
	Heart rate	Onboarding	Numerical
	Respiratory rate	Onboarding	Numerical
	Blood pressure	Onboarding	Numerical
	Conscious level (GCS)	Onboarding	Numerical
	Temperature	Onboarding	Numerical
	NEWS Score	Onboarding	Numerical

Diary Entry (iterates 2-3x daily)	Date and time of recording	Diary	Timestamp
	**Clinical observations:**		
	O2 sats at rest	Diary	Numerical

	O2 sats after exertion	Diary	Numerical
	HR	Diary	Numerical
	Temperature	Diary	Numerical

Diary Aggregate Data (N.B. Derived from Diary entries - not collected in itself)	*Mean pulse*	*Calculated from Diary*	*Numeric*
	Mean O2 sats	*Calculated from Diary*	*Numeric*
	Datetime of lowest O2 Sats	*Calculated from Diary*	*Numeric*
	Value of lowest O2 Sats	*Calculated from Diary*	*Numeric*
	Datetime of highest HR	*Calculated from Diary*	*Numeric*
	Value of highest HR	*Calculated from Diary*	*Numeric*
	Datetime of highest Temp	*Calculated from Diary*	*Numeric*
	Value of highest Temp	*Calculated from Diary*	*Numeric*
	Number of days of fever	*Calculated from Diary*	*Numeric*
	Days on Virtual Ward	*Calculated from Diary*	*Numeric*

In Hospital Data (one for each A&E presentation or hospital admission)	First location on arrival	Hospital EHR / HES	Categorical
	Date and time of arrival	Hospital EHR / HES	Timestamp
	Admitted from A&E	Hospital EHR / HES	Binary
	**Obs on arrival:**		
	O2 sats on air	Hospital EHR	Numeric
	O2 desturation (>3%)	Hospital EHR	Numeric
	Heart rate	Hospital EHR	Numeric
	Respiratory rate	Hospital EHR	Numeric
	Blood pressure	Hospital EHR	Numeric
	Conscious level (GCS)	Hospital EHR	Numeric
	Temperature	Hospital EHR	Numeric
	NEWS Score	Hospital EHR	Numeric
	**Treatment given:**		

	Highest Oxygen Requirement	Hospital EHR	Numeric
	High flow oxygen	Hospital EHR	Binary
	CPAP	Hospital EHR	Binary
	NIV	Hospital EHR	Binary
	Intubation	Hospital EHR	Binary
	level 2 care	Hospital EHR	Binary
	Level 3 care (ITU)	Hospital EHR	Binary
	**Outcomes:**		
	Date and time of discharge or in-hospital d	Hospital EHR / HES	Timestamp
	Discharge destination	Hospital EHR / HES	Categorical
	Length of Stay	*Computed from in-hospital data*	*Numeric*

Mortality	Date of Death	ONS	Date
	Cause(s) of Death	ONS	String

For peer review only

STROBE Statement—checklist of items that should be included in reports of observational studies

	Item No	Recommendation	Page No
**Title and abstract**	1	(*a*) Indicate the study's design with a commonly used term in the title or the abstract	2
		(*b*) Provide in the abstract an informative and balanced summary of what was done and what was found	2
**Introduction**			
Background/rationale	2	Explain the scientific background and rationale for the investigation being reported	3
Objectives	3	State specific objectives, including any prespecified hypotheses	3
**Methods**			
Study design	4	Present key elements of study design early in the paper	4
Setting	5	Describe the setting, locations, and relevant dates, including periods of recruitment, exposure, follow-up, and data collection	4
Participants	6	(*a*) *Cohort study*—Give the eligibility criteria, and the sources and methods of selection of participants. Describe methods of follow-up<br>*Case-control study*—Give the eligibility criteria, and the sources and methods of case ascertainment and control selection. Give the rationale for the choice of cases and controls<br>*Cross-sectional study*—Give the eligibility criteria, and the sources and methods of selection of participants	4
		(*b*) *Cohort study*—For matched studies, give matching criteria and number of exposed and unexposed<br>*Case-control study*—For matched studies, give matching criteria and the number of controls per case	N/A
Variables	7	Clearly define all outcomes, exposures, predictors, potential confounders, and effect modifiers. Give diagnostic criteria, if applicable	4
Data sources/ measurement	8*	For each variable of interest, give sources of data and details of methods of assessment (measurement). Describe comparability of assessment methods if there is more than one group	*4*
Bias	9	Describe any efforts to address potential sources of bias	4,5
Study size	10	Explain how the study size was arrived at	4
Quantitative variables	11	Explain how quantitative variables were handled in the analyses. If applicable, describe which groupings were chosen and why	4,5
Statistical methods	12	(*a*) Describe all statistical methods, including those used to control for confounding	5
		(*b*) Describe any methods used to examine subgroups and interactions	5
		(*c*) Explain how missing data were addressed	5
		(*d*) *Cohort study*—If applicable, explain how loss to follow-up was addressed<br>*Case-control study*—If applicable, explain how matching of cases and controls was addressed<br>*Cross-sectional study*—If applicable, describe analytical methods taking account of sampling strategy	N/A
		(*e*) Describe any sensitivity analyses	N/A

Continued on next page

For peer review only - http://bmjopen.bmj.com/site/about/guidelines.xhtml

**Results**			
Participants	13*	(a) Report numbers of individuals at each stage of study—eg numbers potentially eligible, examined for eligibility, confirmed eligible, included in the study, completing follow-up, and analysed	5
		(b) Give reasons for non-participation at each stage	5
		(c) Consider use of a flow diagram	N/A
Descriptive data	14*	(a) Give characteristics of study participants (eg demographic, clinical, social) and information on exposures and potential confounders	5,14
		(b) Indicate number of participants with missing data for each variable of interest	5
		(c) *Cohort study*—Summarise follow-up time (eg, average and total amount)	N/A
Outcome data	15*	*Cohort study*—Report numbers of outcome events or summary measures over time	
		Case-control study—Report numbers in each exposure category, or summary measures of exposure	
		Cross-sectional study—Report numbers of outcome events or summary measures	*6*
Main results	16	(*a*) Give unadjusted estimates and, if applicable, confounder-adjusted estimates and their precision (eg, 95% confidence interval). Make clear which confounders were adjusted for and why they were included	^
		(*b*) Report category boundaries when continuous variables were categorized	N/A
		(*c*) If relevant, consider translating estimates of relative risk into absolute risk for a meaningful time period	N/A
Other analyses	17	Report other analyses done—eg analyses of subgroups and interactions, and sensitivity analyses	5,6
**Discussion**			
Key results	18	Summarise key results with reference to study objectives	7
Limitations	19	Discuss limitations of the study, taking into account sources of potential bias or imprecision. Discuss both direction and magnitude of any potential bias	8
Interpretation	20	Give a cautious overall interpretation of results considering objectives, limitations, multiplicity of analyses, results from similar studies, and other relevant evidence	7-9
Generalisability	21	Discuss the generalisability (external validity) of the study results	8-9
**Other information**			
Funding	22	Give the source of funding and the role of the funders for the present study and, if applicable, for the original study on which the present article is based	10

*Give information separately for cases and controls in case-control studies and, if applicable, for exposed and unexposed groups in cohort and cross-sectional studies.

Note: An Explanation and Elaboration article discusses each checklist item and gives methodological background and published examples of transparent reporting. The STROBE checklist is best used in conjunction with this article (freely available on the Web sites of PLoS Medicine at http://www.plosmedicine.org/, Annals of Internal Medicine at http://www.annals.org/, and Epidemiology at http://www.epidem.com/). Information on the STROBE Initiative is available at www.strobe-statement.org.

For peer review only

Assessing the Safety of Home Oximetry for Covid-19: A multi-site retrospective observational study.

Journal:	*BMJ Open*
Manuscript ID	bmjopen-2021-049235.R2
Article Type:	Original research
Date Submitted by the Author:	03-Aug-2021
Complete List of Authors:	Clarke, Jonathan; Imperial College London, Centre for Mathematics of Precision Healthcare<br>Flott, Kelsey; Imperial College London, Institute of Global Health Innovation<br>Fernandez Crespo, Roberto; Imperial College London, Institute of Global Health Innovation<br>Ashrafian, Hutan; Imperial College London, Institute of Global Health Innovation<br>Fontana, Gianluca; Imperial College London, Institute of Global Health Innovation<br>Benger, Jonathan; NHS Digital<br>Darzi, Ara; Imperial College London, Institute of Global Health Innovation<br>Elkin, Sarah; Imperial College London, National Heart and Lung Institute
**Primary Subject Heading**:	Respiratory medicine
Secondary Subject Heading:	Diagnostics, General practice / Family practice, Public health, Infectious diseases, Health services research
Keywords:	COVID-19, Telemedicine < BIOTECHNOLOGY & BIOINFORMATICS, PRIMARY CARE, PUBLIC HEALTH, RESPIRATORY MEDICINE (see Thoracic Medicine)

I, the Submitting Author has the right to grant and does grant on behalf of all authors of the Work (as defined in the below author licence), an exclusive licence and/or a non-exclusive licence for contributions from authors who are: i) UK Crown employees; ii) where BMJ has agreed a CC-BY licence shall apply, and/or iii) in accordance with the terms applicable for US Federal Government officers or employees acting as part of their official duties; on a worldwide, perpetual, irrevocable, royalty-free basis to BMJ Publishing Group Ltd ("BMJ") its licensees and where the relevant Journal is co-owned by BMJ to the co-owners of the Journal, to publish the Work in this journal and any other BMJ products and to exploit all rights, as set out in our licence.

The Submitting Author accepts and understands that any supply made under these terms is made by BMJ to the Submitting Author unless you are acting as an employee on behalf of your employer or a postgraduate student of an affiliated institution which is paying any applicable article publishing charge ("APC") for Open Access articles. Where the Submitting Author wishes to make the Work available on an Open Access basis (and intends to pay the relevant APC), the terms of reuse of such Open Access shall be governed by a Creative Commons licence – details of these licences and which Creative Commons licence will apply to this Work are set out in our licence referred to above.

Other than as permitted in any relevant BMJ Author's Self Archiving Policies, I confirm this Work has not been accepted for publication elsewhere, is not being considered for publication elsewhere and does not duplicate material already published. I confirm all authors consent to publication of this Work and authorise the granting of this licence.

For peer review only

Assessing the Safety of Home Oximetry for Covid-19: A multi-site retrospective observational study.

Authors:

Jonathan Clarke[1]

Kelsey Flott[2]

Roberto Crespo[2]

Hutan Ashrafian[2]

Gianluca Fontana[2]

Jonathan Benger[3]

Ara Darzi[2]

Sarah Elkin[4]

Institution:

1. Centre for Mathematics of Precision Healthcare, Imperial College London, London, SW7 2AZ
2. Institute of Global Health Innovation, Imperial College London, London, SW7 2NA
3. NHS Digital, 1 Trevelyan Square, Boar Lane, Leeds, West Yorkshire. LS1 6AE.
4. National Heart and Lung Institute, Imperial College London, Guy Scadding Building, Cale Street, London, SW3 6LY

Word count:

3515

Corresponding Author

Dr Jonathan Clarke

Centre for Mathematics of Precision Healthcare, Imperial College London, London SW7 2AZ

j.clarke@imperial.ac.uk

+44 (0) 7792 690037

For peer review only

Abstract

Objectives

To determine the safety and effectiveness of home oximetry monitoring pathways safe for Covid-19 patients in the English NHS.

Design

Retrospective, multi-site, observational study of home oximetry monitoring for patients with suspected or proven Covid-19

Setting

This study analysed patient data from four Covid-19 home oximetry pilot sites in England across primary and secondary care settings.

Participants

A total of 1338 participants were enrolled in a home oximetry programme across four pilot sites. Participants were excluded if primary care data and oxygen saturations are rest at enrolment were not available. Data from 908 participants was included in the analysis.

Interventions

Home oximetry monitoring was provided to participants with a known or suspected diagnosis of Covid-19. Participants were enrolled following attendance to emergency departments, hospital admission or referral through primary care services.

Results

Of 908 patients enrolled into four different Covid-19 home oximetry programmes in England, 771 (84.9%) had oxygen saturations at rest of 95% or more, and 320 (35.2%) were under 65 years of age and without comorbidities. 52 (5.7%) presented to hospital and 28 (3.1%) died following enrolment, of which 14 (50%) had Covid-19 as a named cause of death. All-cause mortality was significantly higher in patients enrolled after admission to hospital (OR 8.70 [2.53-29.89]), compared to those enrolled in primary care. Patients enrolled after hospital discharge (OR 0.31 [0.15-0.68]) or emergency department presentation (OR 0.42 [0.20-0.89]) were significantly less likely to present to hospital than those enrolled in primary care.

Conclusions

This study find that home oximetry monitoring can be a safe pathway for Covid-19 patients; and indicates increases in risk to vulnerable groups and patients with oxygen saturations < 95% at enrolment, and in those enrolled on discharge from hospital. Findings from this

For peer review only

evaluation have contributed to the national implementation of home oximetry across England.

For peer review only

For peer review only - http://bmjopen.bmj.com/site/about/guidelines.xhtml

Strengths and limitations of this study

- This multisite study examines outcomes and variation between four Covid-19 home oximetry remote monitoring pathways in England.
- It uses linked data from primary and secondary care, alongside mortality data and data collected from the oximetry pathway to understand clinical outcomes and prior risk factors.
- It examines the clinical status of patients enrolled under different home oximetry pathways and examines their rates of hospital attendance and all-cause mortality.
- The study is limited to four sites, and is unable to comment on the outcomes of patients not enrolled into these pathways or on the performance of pathways not included in the study.

For peer review only

For peer review only - http://bmjopen.bmj.com/site/about/guidelines.xhtml

Introduction

Before the outbreak of the Covid-19 pandemic, compelling evidence already existed regarding the role of telemedicine and digital technologies in restructuring how healthcare is delivered, indicating an opportunity to expand the use of virtual pathways [1]. Such evidence suggests that care delivered remotely can, in many circumstances, safely meet patients' clinical needs and personal preferences [1–3]. Specifically, remote monitoring pathways, those that rely on an initial point of contact with the health services followed by continuous monitoring via phone calls, digital or app-based diaries or wearable sensors, have also demonstrated effectiveness, especially when supported by behavioural change models [4–6]. However, the clinical effectiveness, safety and economic utility of this type of monitoring is context-dependent and varies considerably across clinical conditions; therefore, more evidence is required to fully assess their impact [4].

During the Covid-19 pandemic, the UK National Health Service (NHS), and health systems across the world, rapidly adopted novel remote monitoring pathways, many relying on home pulse oximetry [7–9]. These pathways were often initiated quickly in an attempt to proactively manage patients without risking unnecessary patient travel to hospital, but subsequently varied in terms of the devices used, patients included and the method of implementation. The available literature suggests potential for home management of Covid to support a positive patient experience [10–12]. However, while pulse oximetry and trends over time appear to be an effective way of detecting deterioration, the evidence surrounding the safety of oximetry devices, specifically low-cost pocket oximeters, is variable and more research is required to understand what oxygen saturation thresholds should trigger a patient to seek in-person care [13,14]. Overall, the literature surrounding whether home oximetry monitoring for Covid-19 patients is safe remains inconclusive [9,15].

In the UK, NHS England/Improvement, the body responsible for improved delivery of NHS care, in partnership with NHS Digital and Imperial College London, set out to understand, quantitatively, whether home oximetry monitoring was a safe clinical pathway to implement nationally. Following the first UK peak of Covid-19, in Spring 2020, pilots of home oximetry monitoring tested a system-wide approach to the early detection of Covid-19 in the community. As part of this pilot, a rapid evaluation was conducted to determine whether home oximetry monitoring was a safe clinical pathway. Based on existing literature, the initial hypothesis was that early recognition, escalation, admission and treatment could save lives in Covid-19 and would be a safe approach to adopt. In December 2020, informed by the findings of this study, NHS England and NHS Improvement recommended that all Clinical Commissioning Groups (CCGs) put in place a Covid-19 home oximetry service. At the time of writing, more than twenty thousand patients in England have been treated on similar pathways to those included in this study [16].

Aims & Objectives

This study aimed to answer the research question: *Are home oximetry monitoring pathways safe and effective for Covid-19 patients in the English NHS?*

The primary objective was to determine whether patients suffered any adverse consequences as a result of home oximetry monitoring. The secondary objective was to explore whether the recommendations relating to oxygen saturation that were published in June 2020 in the NHS Covid-19 assessment pathway were correct. The final objective was to contribute to a

For peer review only - http://bmjopen.bmj.com/site/about/guidelines.xhtml

recommendation to NHS England/Improvement regarding the safety and suitability of home oximetry monitoring as a national Covid-19 pathway.

Methods

Setting & Design

To answer this question, Imperial College NHS Healthcare Trust, NIHR Imperial Patient Safety Translational Research Centre (PSTRC) at the Institute of Global Health Innovation (IGHI) at Imperial College London, working in partnership with NHS Digital, assessed existing evidence concerning home oximetry monitoring for respiratory conditions and analysed prospective data collected from four sites.

This was a retrospective, multi-site, observational study of home oximetry monitoring for patients with suspected or proven Covid-19 in England during Summer 2020, including an analysis of patient data from four pilot sites: North West London, Slough, South Tees and Watford. Sites varied in how patients were enrolled onto pathways; from primary care, after a presentation to the emergency department or following a hospital admission, or a combination of these. In each site, patients were enrolled onto the oximetry pathway and were provided with a pulse oximeter to measure their oxygen saturations and heart rate over time. These recordings, in addition to a patient's symptoms and perceived overall wellbeing were communicated to their nominated healthcare professional (either a general practitioner, practice nurse or hospital doctor) through a combination of app-based platforms and paper diaries. The frequency of recording varied between pathways, from daily to several times per day. In the case of clinical deterioration or other concerns, patients were able to contact their nominated healthcare professional for advice and escalation of care could be initiated as appropriate. While services may vary between sites, the current Standard Operating Procedure for Covid-19 home oximetry services in England can be accessed on the NHS Digital website.[17]

Quantitative analysis was conducted in the IGHI's Big Data and Analytical Unit (BDAU). The BDAU provide a fully certified ISO 27001:2013 research environment within Imperial College and is fully compliant with NHS IG Toolkit Level 3 (EE133887). The work was conducted as a service evaluation, as institutional Research Governance deemed that it did not require further ethics committee approval. Information Governance approval was obtained and a data sharing agreement established (DARS-NIC-396113-N9L4L-v1.2). Analysis took place within the Imperial College London Big Data and Analytical Unit.

Information & Data

Four separate datasets linked by a pseudonymised patient identifier were provided by NHS Digital for the express purpose of this evaluation. A full list of variables collected is included in **Supplement 1** and summarised below:

Primary Care Records: Data pertaining to patient demographics and clinical comorbidities were obtained from the General Practice Extraction Service (GPES) Data for Pandemic Planning and Research (GDPPR) for all patients enrolled into a home oximetry monitoring programme at one of the four sites. Records were available from the start of a patient's primary care record in the practice to the date of data transfer (25th Sept 2020).

For peer review only - http://bmjopen.bmj.com/site/about/guidelines.xhtml

Home oximetry Monitoring Records: For each patient enrolled into a home oximetry monitoring programme at the four sites, data were collected from individual home oximetry monitoring providers consisting of the date of enrolment, oxygen saturations at rest at enrolment and the clinical pathway to which they were enrolled.

Hospital records: The dates, outcomes of Accident and Emergency presentations and admissions to hospital for patients while enrolled in the home oximetry programme were obtained from case note review by participating sites. Data were returned by sites in mid-September 2020.

Mortality records: The date and causes (as ICD-10 codes) of death were provided for each patient whose death had been recorded by the Office for National Statistics after enrolment to the home oximetry programme until the date of data transfer (25th Sept 2020).

For peer review only

Analysis & Statistical Procedure

Data were linked according to the pseudonymised patient identifier. A single cohort of patients was identified where GDPPR data and oxygen saturations at rest at enrolment were available. The demographic and clinical characteristics of this population where described and differences in patient and clinical characteristics were compared between routes of enrolment using pairwise Fisher's Exact Tests for count data and Mann Whitney U-tests for non-parametric continuous data.

The likelihood of a patient presenting to hospital at least once following enrolment to the home oximetry monitoring programme was examined using univariable and multivariable logistic regression models. The likelihood of all-cause mortality following enrolment to the home oximetry monitoring programme was also examined using univariable and multivariable logistic regression models. Frequent absence of dates of enrolment or dates of hospital admission precluded formal temporal evaluation of time to hospital presentation or mortality.

Results

A total of 1338 patients were recorded as being enrolled into the home oximetry programme at one of the four pilot sites. Of these, GDPPR records were available for 1242 patients (92.8%). Of these patients, a recorded oxygen saturation level at enrolment onto the home oximetry was present for 908 patients (73.1%).

Comparison of Enrolment Pathways

The characteristics of the included population, overall and according to the route of enrolment, are described in **Table 1**. 302 patients were enrolled from primary care (33.4%), 342 from A&E (37.9%) and 259 following discharge from hospital (28.9%). Route of enrolment was missing for five patients (0.6%).

Oxygen saturations at enrolment were significantly lower in those enrolled on discharge from hospital (96%) than through A&E (97%) or primary care (98%) (MWU, $p < 0.0001$). No difference was observed between primary care and A&E pathways (MWU, $p = 0.0852$). Similarly, there was no significant difference in the proportion of patients enrolled with oxygen

For peer review only - http://bmjopen.bmj.com/site/about/guidelines.xhtml

saturations of 95% or more in primary care (92.0%) or A&E (91.8%) (Fishers Exact Test, $p = 1$), however only 67.6% of patients enrolled after hospital discharge had oxygen saturations of 95% or more (pairwise Fishers Exact Tests, $p < 0.0001$).

Patients enrolled through A&E were most likely to have no comorbidities (47.1%) compared to primary care (37.4%) and hospital discharge (27.0). Primary care patients were less likely to have comorbidities than those enrolled after hospital discharge (pairwise Fishers Exact Tests, $p < 0.05$ in all cases). Patients enrolled through A&E were younger (median age = 50 years) than those enrolled in primary care (55 years) and on hospital discharge (63 years) (MWU, $p < 0.0001$ in all cases).

Presentation to Hospital

A total of 69 presentations to hospital were made by 52 patients (5.7%) after enrolment. 40 of these patients (76.9%) were admitted to hospital, and 8 patients (15.4%) presented more than once. The proportion of patients presenting to hospital according to age group and number of clinical comorbidities is shown in **Figure 1** and **Figure 2**.

3.1% of patients under the age of 65 years and without comorbidities presented to hospital, compared to 5% of those under 65 with comorbidities and 9.7% of those aged 65 and over. Similarly, 5.3% of patients with oxygen saturations of 95% or more at enrolment presented to hospital, compared to 6.1% of those with oxygen saturations of 93-94% and 10.9% of those with oxygen saturations less than 93% at enrolment (**Table 2**).

In univariable logistic regression models, presentation to hospital was significantly more likely in older patients and those with more clinical comorbidities, while those initiated on the home oximetry through A&E or on discharge from hospital were less likely to re-present to hospital (**Table 3**). In the multivariable model, increasing age (OR 1.03, $p = 0.018$) was associated with significantly higher odds of presentation to hospital, while those initiated through A&E (OR 0.42, $p = 0.024$) and following discharge from hospital (OR 0.31, $p = 0.003$) were significantly less likely to present to hospital (**Table 3**).

All-cause mortality

A total of 28 patients (3.1%) died of any cause following enrolment. 14 of these patients (50%) had Covid-19 as a named cause of death (ICD-10 code U07.1), and 12 (42.9%) had Covid-19 as the underlying cause of death. All-cause mortality by age group and number of clinical comorbidities is shown in **Figure 1** and **Figure 2**. Only patients with one or more comorbidities died, while 17 (60.1%) patients who died had four or more clinical comorbidities.

None of the 320 patients under the age of 65 and without comorbidities included in the study died during the study period, compared to 0.9% of those under 65 with comorbidities and 9.3% of those aged 65 and over. 2.6% of patients with oxygen saturations of 95% or more at enrolment died during the study period, compared to 6.1% of those with oxygen saturations of 93-94% and 5.5% of those with oxygen saturations less than 93% at enrolment (**Table 2**).

For peer review only - http://bmjopen.bmj.com/site/about/guidelines.xhtml

For peer review only

In univariable logistic regression models, all-cause mortality was significantly more likely in older patients, those of BAME ethnicity, with more clinical comorbidities, who were overweight or obese and those initiated on the home oximetry following discharge from hospital (**Table 4**). In the multivariable model, increasing age (OR 1.08, p = 0.000), more clinical comorbidities (OR 1.45, p = 0.009), being overweight or obese (OR 4.83, p = 0.002) and being initiated on the home oximetry following discharge from hospital (OR 8.70, p = 0.001) were associated with significantly higher odds of all-cause mortality (**Table 4**).

Discussion

Statement of key findings

In terms of clinical safety, only 5.7% of patients presented to hospital after enrolment. The likelihood of presenting to hospital increased with age, but was not significantly associated with ethnicity, number of clinical comorbidities, obesity or oxygen saturations at enrolment. However, all-cause mortality was significantly more likely with increasing age, number of clinical comorbidities and obesity. All-cause mortality was also higher for those patients who initiated the pathway after discharge from hospital. Finally, most patients using home oximetry monitoring were of low clinical severity on initiation and neither died nor presented to hospital during the study period. Therefore, the Covid-19 assessment pathway published in June 2020 is clinically appropriate, and its recommendations regarding oxygen saturation thresholds are correct.

While hospital presentation and mortality were associated with lower oxygen saturations at enrolment, this was not a statistically significant relationship after controlling for other patient factors. Therefore, although oxygen saturation at enrolment may provide an important tool by which to stratify clinical severity, such assessments must also incorporate a wider understanding of factors determining outcome including age, obesity and clinical comorbidities.

None of the 320 participants aged less than 65 and without comorbidities died during the study period. These individuals accounted for 33.3% of all participants which suggests that during the pilots a large proportion of the participants were low risk at enrolment and did not suffer any adverse outcome. Wider implementation should therefore focus on higher risk groups to ensure efficient use of limited health and care resources.

Statistically significant variation in the characteristics of patients, clinical severity of illness, rates of hospital presentation and all-cause mortality were observed between the three routes of enrolment. Patients enrolled following a hospital admission had 8.7 times higher odds of all cause mortality than patients enrolled in primary care after adjusting for age, sex, ethnicity, obesity, clinical comorbidities and oxygen saturation at enrolment. Conversely, compared to those enrolled in primary care, patients enrolled after an A&E presentation or hospital admission had significantly lower odds of further presentation to hospital after enrolment. Collectively, this indicates differences in patient characteristics and the risk of adverse events according to the route of enrolment. Those enrolled in primary care are generally the least unwell and go on to have lower rates of hospital presentation and mortality. The finding that

For peer review only - http://bmjopen.bmj.com/site/about/guidelines.xhtml

patients enrolled in A&E or following hospital admission have lower rates of hospital presentation may indicate reluctance on the part of patients to return to hospital during the same period of illness. Patients enrolled following hospital discharge appear to be at particularly high risk of mortality and may therefore represent a patient population that should either be offered more intensive monitoring or alternative approaches to reducing the risk of subsequent mortality.

Following enrolment more patients aged 90 years and over died during the study period than presented to hospital, while in younger age groups hospital presentations outnumbered all-cause mortality (Figure 1). A similar trend was observed according to increasing clinical comorbidities (Figure 2). This is likely to reflect circumstances in which hospital admission is not in accordance with a patient's wishes or considered clinically appropriate, and end-of-life care is therefore initiated in a person's usual place of residence.

Taken together, our results support the hypothesis that oximetry monitoring is a safe pathway for Covid-19 patients. This was reported to NHS England and Improvement in October 2020.

Strengths & Limitations

The primary strength of this study is its system-level and patient-level impact, since this work evaluated a real-world pilot of a new clinical pathway. The project team achieved an integrated partnership between academics, clinicians and policy makers. This enabled a direct pipeline from evidence generation to policy decision making; moreover, the efficiency with which the work was conducted was nationally important, as it was required to inform how the NHS would use home oximetry monitoring during the next wave of the pandemic. Impact was achieved through the unique composition of the evaluation team as well as the close relationship the team held to NHS leaders in home oximetry monitoring. The method for this work required novel data reporting from the pilot sites to the evaluation team and this was ensured through close collaboration with the individual sites.

The most prevalent limitation related to data quality and completeness. The pilot sites varied in their routes of referral and in the clinical severity of the patients they enrolled. Furthermore, there was heterogeneity in the actual intervention across the sites, as some enrolled patients with suspected Covid-19, while others included patients who had received hospital treatment for Covid-19 and were subsequently discharged. Due to the rapid timescales of this evaluation and the pressured environment in which it took place, mandating full completeness in the data submitted was not possible, and the resulting dataset had a considerable amount of missing data. The lack of longitudinal oxygen saturations data precluded temporal analysis of patient trajectories and identification of deterioration in clinical status. While such an evaluation would have been valuable, it was not possible within the available dataset. In addition to oxygen saturations, other physiological parameters, in including respiratory rate, may be important indicators of clinical severity in patients with Covid-19 [18]. In this study, only oxygen saturations were available which precluded analysis of clinical severity according to a broader range of physiological parameters.

In addition, the absence of precise temporal recording of data and an inability to complete individual case note reviews precluded a more detailed evaluation of whether deaths occurred

For peer review only - http://bmjopen.bmj.com/site/about/guidelines.xhtml

as a result of Covid-19 infection, or another cause entirely. Finally, this was not a controlled study, which prevented a comparative analysis. Due to these limitations and the relatively low community prevalence of Covid-19 during the pilot, our findings are not necessarily generalisable to future waves of Covid-19. Additionally, while many initial oxygen saturations are likely to be taken in the presence of a healthcare professional, we are unable to determine whether these readings are taken correctly and may therefore provide inappropriate estimates of clinical acuity.

Comparison to other studies

While research evidence regarding Covid-19 home oximetry is still in its infancy, this study reflects similar findings in the published literature. One systematic review of home monitoring for Covid-19 acknowledges inconclusive evidence about effectiveness and safety. While this study could not conclusively determine the clinical effectiveness of the pilots, it was able to demonstrate non-inferiority in terms of safety with traditional hospital management of Covid-19.

Furthermore, this study indicates the potential for these technologies to support pandemic management in line with expert opinions published in the literature [19]. The findings of this work are consistent with existing grey literature suggesting the utility of home oximetry monitoring for Covid-19 [8,20]. This study specifically assessed the safety of home oximetry for Covid-19 and makes policy recommendations not present in existing studies which tend to focus more on patient experience and potential clinical effectiveness. These findings echo the findings of a previous systematic review indicating a paucity of studies directly addressing patient safety in telemedicine [21]. Identifying the potential patient safety risks arising from the national home oximetry programme is an area of ongoing qualitative and quantitative research as part of the national Covid-19 Oximetry at Home Programme [20].

Implications for Policy

This work was explicitly designed to answer relevant questions in advance of the national implementation of a new clinical pathway for Covid-19. It confirms the appropriateness of existing policies and that safety netting approaches are congruent with findings.

This study provides information as to how the pathway should function including the thresholds at which people should be enrolled into home oximetry monitoring programmes, how long they should continue in home oximetry monitoring and what sorts of technology is required to support home oximetry monitoring for Covid-19.

Furthermore, the practicalities of running this study revealed important considerations for the ongoing evaluation of home oximetry monitoring pathways for Covid-19. It is clear from the study that it is only possible to measure the safety of home oximetry monitoring with the robust and rigorous collection of data from healthcare providers as well as any third party app-based providers. This data flow should continue during national implementation to continuously measure safety under different conditions and levels of organisational pressure.

Conclusion

For peer review only - http://bmjopen.bmj.com/site/about/guidelines.xhtml

Advancing the evidence base regarding the safety of home oximetry monitoring for Covid-19 patients is of considerable importance as many health systems face new waves of the pandemic worldwide. This study reveals, via a real-world pilot evaluation, that home oximetry monitoring can be a safe pathway for Covid patients; however, substantial research is needed to understand its clinical effectiveness across patient populations. This study was limited by complex and incomplete data as well as variation in intervention designs; a product of the pandemic context within which it was undertaken. However, it did provide initial evidence of the appropriate clinical thresholds and patient characteristics for home oximetry. It also indicated increases in risk to vulnerable groups and patients with oxygen saturations below 95% at enrolment.

Findings from this evaluation have contributed to the national implementation of home oximetry across England, and further work will be undertaken to evaluate clinical effectiveness and any inequalities in terms of access to, and inclusion in, the new pathway.

For peer review only

Funding

This research was supported by the National Institute for Health Research (NIHR) Imperial Patient Safety Translational Research Centre (PSTRC). The views expressed are those of the authors and not necessarily those of the NHS, the NIHR or the Department of Health and Social Care. JC acknowledges support from the Wellcome Trust (215938/Z/19/Z).

Conflicts of Interest

All authors have completed the ICMJE uniform disclosure form at www.icmje.org/coi_disclosure.pdf and have no competing interests to declare.

Acknowledgements

The research team would like to thank the teams at NHS Digital and NHS England/Improvement as well as the site leads from the home oximetry pilot sites.

Data Sharing Statement

Patient-level pseudonymised data were obtained from NHS Digital for the specific purpose of the evaluation. Aggregate findings are presented in this study. Patient-level data may be obtained through application and review by NHS Digital.

Contribution Statement

JC, KF and RC were involved in all aspects of the study. HA, GF, JB, AD and SE were involved in the conceptualisation of the study, interpretation of findings and in the reviewing and editing of the draft. JC has had access to all the data in the study and all authors had final responsibility for the decision to submit for publication. JC attests that all listed authors meet authorship criteria and that no others meeting the criteria have been omitted.

Copyright Statement

The Corresponding Author has the right to grant on behalf of all authors and does grant on behalf of all authors, a worldwide licence to the Publishers and its licensees in perpetuity, in all forms, formats and media (whether known now or created in the future), to i) publish, reproduce, distribute, display and store the Contribution, ii) translate the Contribution into other languages, create adaptations, reprints, include within collections and create summaries, extracts and/or, abstracts of the Contribution, iii) create any other derivative work(s) based on the Contribution, iv) to exploit all subsidiary rights in the Contribution, v) the inclusion of electronic links from the Contribution to third party material where-ever it may be located; and, vi) licence any third party to do any or all of the above.

Patient and Public Involvement Statement

Patients and/or the public were not involved in the design, or conduct, or reporting, or dissemination plans of this research.

For peer review only - http://bmjopen.bmj.com/site/about/guidelines.xhtml

Transparency Declaration

JC affirms that the manuscript is an honest, accurate, and transparent account of the study being reported; that no important aspects of the study have been omitted; and that any discrepancies from the study as originally planned (and, if relevant, registered) have been explained.

Ethics Approval Statement

The work was conducted as a service evaluation, as the Imperial College Research Ethics Committee deemed that it did not require further ethics committee approval.

For peer review only

Citations

1 Greenhalgh T, Vijayaraghavan S, Wherton J, *et al.* Virtual online consultations: Advantages and limitations (VOCAL) study. *BMJ Open* 2016;**6**:e009388. doi:10.1136/bmjopen-2015-009388

2 Personalised Health and Care 2020 Using Data and Technology to Transform Outcomes for Patients and Citizens A Framework for Action. 2014.

3 Joshi M, Ashrafian H, Arora S, *et al.* Digital alerting and outcomes in patients with sepsis: Systematic review and meta-analysis. J. Med. Internet Res. 2019;**21**:e15166. doi:10.2196/15166

4 Noah B, Keller MS, Mosadeghi S, *et al.* Impact of remote patient monitoring on clinical outcomes: an updated meta-analysis of randomized controlled trials. npj Digit. Med. 2018;**1**:1–12. doi:10.1038/s41746-017-0002-4

5 Kim YN, Shin DG, Park S, *et al.* Randomized clinical trial to assess the effectiveness of remote patient monitoring and physician care in reducing office blood pressure. *Hypertens Res* 2015;**38**:491–7. doi:10.1038/hr.2015.32

6 Iqbal FM, Joshi M, Davies G, *et al.* Design of the pilot, proof of concept REMOTE-COVID trial: remote monitoring use in suspected cases of COVID-19 (SARS-CoV-2). *Pilot Feasibility Stud* 2021;**7**:1–7. doi:10.1186/s40814-021-00804-4

7 Medina M, Babiuch C, Card M, *et al.* Home monitoring for COVID-19: Posted April 18, 2020. *Cleve Clin J Med* 2020;**87**:1–4. doi:10.3949/CCJM.87A.CCC028

8 Coronavirus » Pulse oximetry to detect early deterioration of patients with COVID-19 in primary and community care settings. https://www.england.nhs.uk/coronavirus/publication/pulse-oximetry-to-detect-early-deterioration-of-patients-with-covid-19-in-primary-and-community-care-settings/ (accessed 12 May 2021).

9 Gerke S, Shachar C, Chai PR, *et al.* Regulatory, safety, and privacy concerns of home monitoring technologies during COVID-19. *Nat Med* 2020;**26**:1176–82. doi:10.1038/s41591-020-0994-1

10 Morgan AU, Balachandran M, Do D, *et al.* Remote Monitoring of Patients with Covid-19: Design, implementation, and outcomes of the first 3,000 patients in COVID Watch. *Nejm Catal Innov Care Deliv* doi:10.1056/CAT.20.0342

11 Taiwo O, Ezugwu AE. Smart healthcare support for remote patient monitoring during covid-19 quarantine. *Informatics Med Unlocked* 2020;**20**:100428. doi:10.1016/j.imu.2020.100428

12 Watson AR, Wah R, Thamman R. The value of remote monitoring for the COVID-19 pandemic. *Telemed e-Health* 2020;**26**:1110–2. doi:10.1089/tmj.2020.0134

13 Levitan RM. Pulse Oximetry as a Biomarker for Early Identification and Hospitalization of COVID-19 Pneumonia. Acad. Emerg. Med. 2020;**27**:785–6. doi:10.1111/acem.14052

14 Luks AM, Swenson ER. Pulse oximetry for monitoring patients with COVID-19 at home potential pitfalls and practical guidance. Ann. Am. Thorac. Soc. 2020;**17**:1040–6. doi:10.1513/AnnalsATS.202005-418FR

15 Vindrola-Padros C, Singh KE, Sidhu MS, *et al.* Remote home monitoring (virtual wards) during the COVID-19 pandemic: A systematic review. medRxiv. 2020;:2020.10.07.20208587. doi:10.1101/2020.10.07.20208587

For peer review only

16 COVID Oximetry @home - Management Information File 6th May 2021 - NHS Digital. https://digital.nhs.uk/data-and-information/supplementary-information/2021/covid-oximetry-at-home---management-information-file-6-may-2021 (accessed 18 May 2021).

17 NHS Digital. Coronavirus » Novel coronavirus (COVID-19) standard operating procedure: COVID Oximetry @home. https://www.england.nhs.uk/coronavirus/publication/novel-coronavirus-covid-19-standard-operating-procedure-covid-oximetry-home/ (accessed 19 Aug 2021).

18 Jouffroy R, Jost D, Prunet B. Prehospital pulse oximetry: a red flag for early detection of silent hypoxemia in COVID-19 patients. *Crit Care* 2020;**24**:313. doi:10.1186/s13054-020-03036-9

19 Zhao M, Wasfy JH, Singh JP. Sensor-aided continuous care and self-management: implications for the post-COVID era. Lancet Digit. Heal. 2020;**2**:e632–4. doi:10.1016/S2589-7500(20)30220-X

20 COVID Oximetry @home - digital and data services - NHS Digital. https://digital.nhs.uk/coronavirus/covid-oximetry-at-home-digital-and-data-services (accessed 12 May 2021).

21 Guise V, Anderson J, Wiig S. Patient safety risks associated with telecare: A systematic review and narrative synthesis of the literature. *BMC Health Serv Res* 2014;**14**:1–15. doi:10.1186/s12913-014-0588-z

For peer review only

Figures:

Figure 1: Percentage all-cause mortality and presentation to hospital according to patient age

Figure 2: Percentage all-cause mortality and presentation to hospital according to number of clinical comorbidities

For peer review only

Tables:

		Primary Care	Accident & Emergency	Inpatient discharge	Overall
Total Patients		302	342	259	908
Female		180 (59.6)	205 (59.9)	120 (46.3)	508 (55.9)
Median Age (years)		55	50	63	54
Ethnicity	White	113 (37.4)	160 (46.8)	140 (54.1)	415 (45.7)
	Black, Asian or Minority Ethnic	82 (27.2)	103 (30.1)	59 (22.8)	244 (26.9)
	Not recorded	107 (35.4)	79 (23.1)	60 (23.2)	249 (27.4)
Overweight or obese		108 (35.8)	189 (55.3)	160 (61.8)	458 (50.4)
Median oxygen saturations		98	97	96	97
Clinical severity based on oxygen saturations	Mild (≥ 95%)	278 (92.1)	314 (91.8)	175 (67.6)	771 (84.9)
	Moderate (93-94%)	17 (5.6)	22 (6.4)	42 (16.2)	82 (9.0)
	Severe (≤ 92%)	7 (2.3)	6 (1.8)	42 (16.2)	55 (6.1)
Number of clinical comorbidities	0	113 (37.4)	161 (47.1)	70 (27.0)	346 (38.1)
	1	75 (24.8)	105 (30.7)	70 (27.0)	252 (27.7)
	2	42 (13.9)	45 (13.2)	44 (17.0)	131 (14.4)
	3+	72 (23.8)	31 (9.1)	75 (29.0)	179 (19.7)
Hypertension		102 (33.8)	65 (19.0)	111 (42.9)	278 (30.8)
Depression		86 (28.5)	84 (24.6)	72 (27.8)	242 (26.8)
Asthma		75 (24.8)	83 (24.3)	49 (18.9)	207 (22.9)
Steroid use		87 (28.8)	62 (18.1)	54 (20.8)	203 (22.5)
Diabetes mellitus		56 (18.5)	43 (12.6)	63 (24.3)	162 (17.9)
Pregnancy		37 (12.3)	74 (21.6)	23 (8.9)	134 (14.8)
Chronic heart disease		43 (14.2)	35 (10.2)	55 (21.2)	133 (14.7)
Mild frailty		41 (13.6)	17 (5.0)	42 (16.2)	100 (11.1)
Moderate frailty		45 (14.9)	8 (2.3)	43 (16.6)	96 (10.6)
Chronic respiratory disease		39 (12.9)	17 (5.0)	35 (13.5)	91 (10.1)
Chronic kidney disease		32 (10.6)	17 (5.0)	41 (15.8)	90 (10.0)
Cancer		32 (10.6)	23 (6.7)	23 (8.9)	78 (8.6)
Hypothyroidism		29 (9.6)	22 (6.4)	17 (6.6)	68 (7.5)
Hypercholesterolemia		30 (9.9)	14 (4.1)	22 (8.5)	66 (7.3)
Chronic neurological disease		28 (9.3)	7 (2.0)	25 (9.7)	60 (6.6)
Severe frailty		*	*	*	53 (5.9)
Stroke		*	*	*	46 (5.1)
Immunosuppression drug		8 (2.6)	12 (3.5)	7 (2.7)	27 (3.0)
Dementia		*	*	*	25 (2.8)
Mental illness		8 (2.6)	6 (1.8)	8 (3.1)	22 (2.4)
Epilepsy		*	*	*	20 (2.2)
Peripheral vascular disease		*	*	*	13 (1.4)
Autoimmune disease		*	*	*	13 (1.4)
Chronic liver disease		*	*	*	9 (1.0)
Learning disability		*	*	*	6 (0.7)

For peer review only - http://bmjopen.bmj.com/site/about/guidelines.xhtml

Table 1: Characteristics of the study population, overall and according to the route of enrolment. NB: Route of enrolment was missing for 5 of 908 patients. Comorbidity case numbers are not presented for routes of enrolment where one or more route of enrolment involved 5 of fewer patients.

For peer review only

For peer review only - http://bmjopen.bmj.com/site/about/guidelines.xhtml

Table 2: Number of patients, frequency of hospital presentation and all-cause mortality according to risk groups and oxygen saturations at enrolment

Outcome	Risk Group	Oxygen Saturations Severity			
		Mild (≥ 95%)	Moderate (93-94%)	Severe (≤ 92%)	Overall
Total Patients	>= 65 years	203 (75.5)	37 (13.8)	29 (10.8)	269 (29.6)
	< 65 years with comorbidities	277 (86.8)	29 (9.1)	13 (4.1)	319 (35.1)
	< 65 years without comorbidities	291 (90.9)	16 (5.0)	13 (4.1)	320 (35.2)
	Overall	771 (84.9)	82 (9.0)	55 (6.1)	908 (100.0)
Hospital presentation	>= 65 years	18 (8.9)	3 (8.1)	5 (17.2)	26 (9.7)
	< 65 years with comorbidities	13 (4.7)	2 (6.9)	1 (7.7)	16 (5.0)
	< 65 years without comorbidities	10 (3.4)	0 (0.0)	0 (0.0)	10 (3.1)
	Overall	41 (5.3)	5 (6.1)	6 (10.9)	52 (5.7)
All-cause mortality	>= 65 years	18 (8.9)	4 (10.8)	3 (10.3)	25 (9.3)
	< 65 years with comorbidities	2 (0.7)	1 (3.4)	0 (0.0)	3 (0.9)
	< 65 years without comorbidities	0 (0.0)	0 (0.0)	0 (0.0)	0 (0.0)
	Overall	20 (2.6)	5 (6.1)	3 (5.5)	28 (3.1)

For peer review only

Table 3: Univariable and multivariable binary logistic regression output for presentation to hospital following enrolment to the home oximetry pathway

		Univariable			Multivariable		
		OR	p-value	95% CI of OR	OR	p-value	95% CI of OR
Age		1.03	**0.000**	1.01 - 1.05	1.03	**0.018**	1.00 - 1.05
Ethnicity	White	reference			reference		
	Black, Asian and Minority Ethnic	1.22	0.544	0.64 - 2.32	1.57	0.198	0.79 - 3.10
	Not recorded	0.75	0.447	0.36 - 1.57	0.95	0.912	0.42 - 2.17
Number of comorbidities		1.25	**0.004**	1.08 - 1.45	1.07	0.503	0.88 - 1.30
Overweight or Obese		0.67	0.175	0.38 - 1.19	0.55	0.069	0.29 - 1.05
Clinical severity based on oxygen saturations	Mild (≥ 95%)	reference			reference		
	Moderate (93-94%)	1.16	0.766	0.44 - 3.01	1.08	0.885	0.40 - 2.92
	Severe (≤ 92%)	2.18	0.091	0.88 - 5.39	2.35	0.096	0.86 - 6.46
Enrolment Pathway	Primary care	reference			reference		
	Accident & Emergency	0.37	**0.005**	0.18 - 0.74	0.42	**0.024**	0.20 - 0.89
	Inpatient	0.49	**0.049**	0.25 - 0.98	0.31	**0.003**	0.15 - 0.68

For peer review only - http://bmjopen.bmj.com/site/about/guidelines.xhtml

Table 4: Univariable and multivariable binary logistic regression output for all-cause mortality following enrolment to the home oximetry pathway

		Univariable			Multivariable		
		OR	p	95% CI of OR	OR	p	95% CI of OR
Age		1.12	**0.000**	1.08 - 1.16	1.08	**0.000**	1.03 - 1.13
Ethnicity	White	reference			reference		
	Black, Asian and Minority Ethnic	0.16	**0.015**	0.04 - 0.70	0.37	0.235	0.07 - 1.90
	Not recorded	0.49	0.129	0.19 - 1.23	0.61	0.371	0.21 - 1.79
Number of comorbidities		1.97	**0.000**	1.63 - 2.40	1.45	**0.009**	1.09 - 1.92
Overweight or Obese		3.87	**0.004**	1.56 - 9.64	4.83	**0.002**	1.74 - 13.37
Clinical severity based on oxygen saturations	Mild (≥ 95%)	reference			reference		
	Moderate (93-94%)	2.43	0.083	0.89 - 6.68	1.11	0.864	0.34 - 3.67
	Severe (≤ 92%)	2.16	0.224	0.62 - 7.53	0.65	0.574	0.14 - 2.93
Enrolment Pathway	Primary care	reference			reference		
	Accident &Emergency	0.66	0.587	0.15 - 2.97	3.40	0.157	0.62 - 18.55
	Inpatient	6.57	**0.001**	2.23 - 19.41	8.70	**0.001**	2.53 - 29.89

For peer review only - http://bmjopen.bmj.com/site/about/guidelines.xhtml

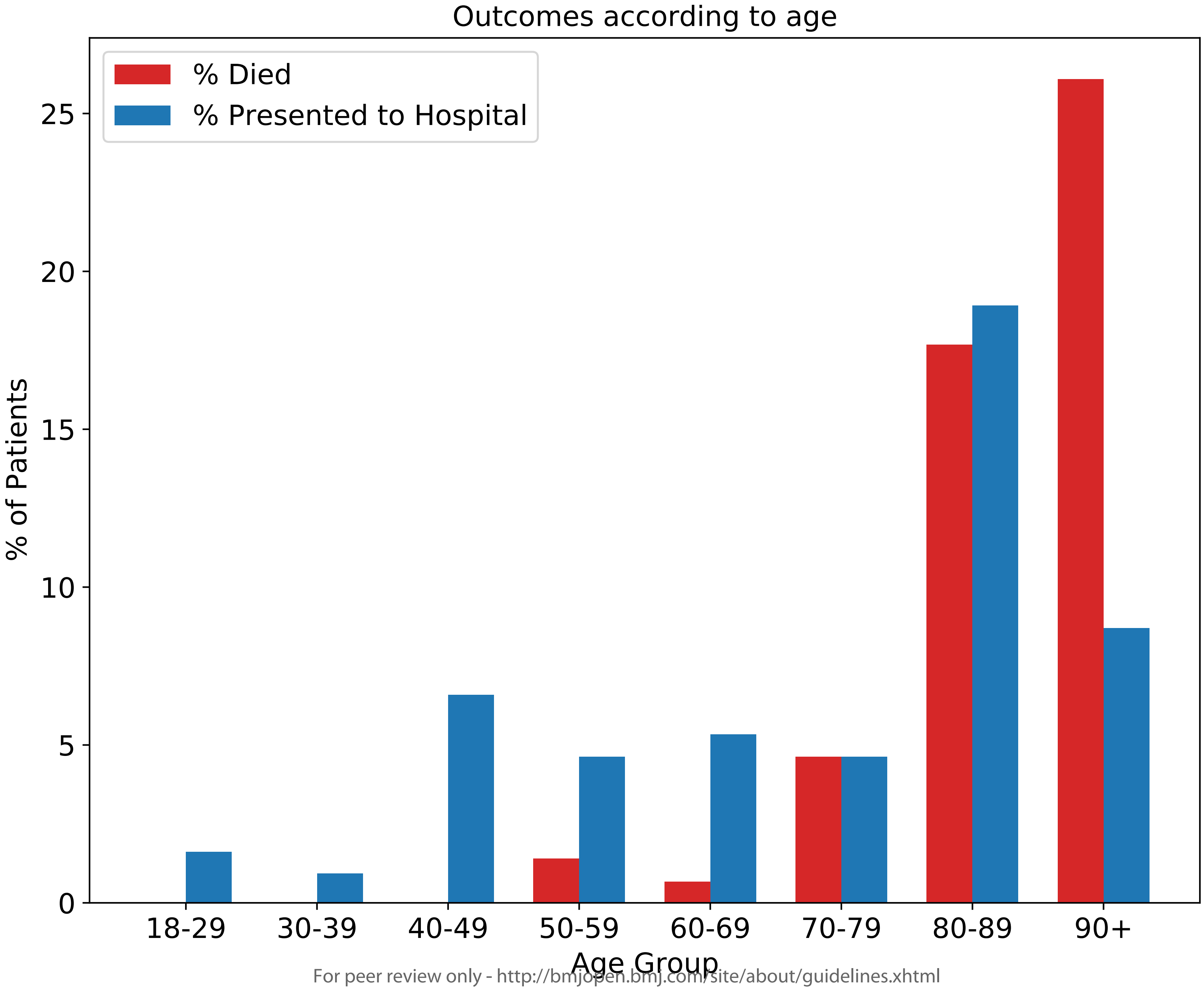
Outcomes according to age
% Died
% Presented to Hospital
% of Patients
0
5
10
15
20
25
18-29
30-39
40-49
50-59
60-69
70-79
80-89
90+
Age Group

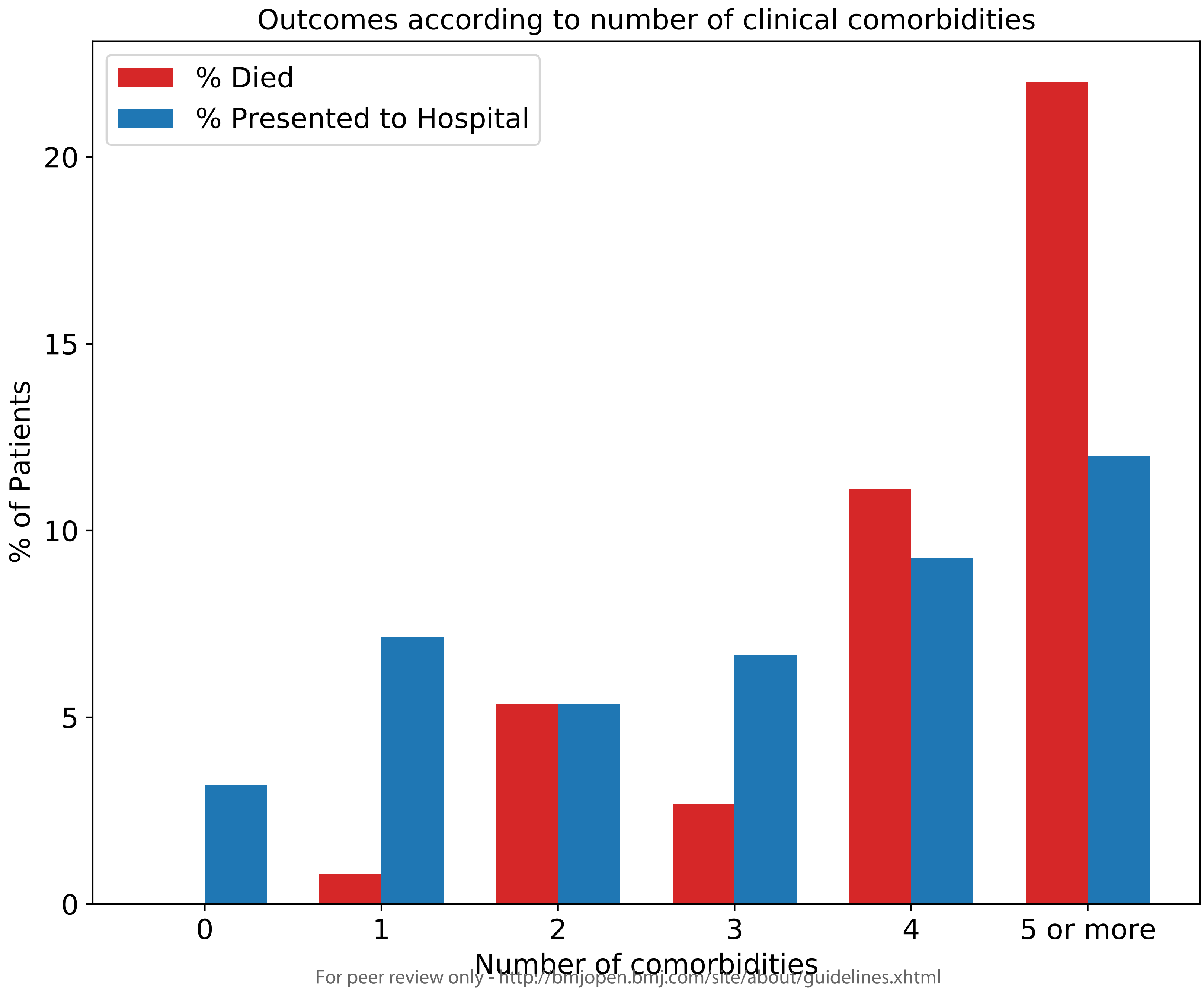
Outcomes according to number of clinical comorbidities
% Died
% Presented to Hospital
% of Patients
0
5
10
15
20
0
1
2
3
4
5 or more
Number of comorbidities

Category	Variable	Source	Data Type
Baseline Data	Age	PDS	Numeric
	Ethnicity	PDS	Categorical
	Sex	PDS	Categorical
	post code	PDS	String
	occupation	Onboarding	Categorical /string
	Registered GP Practice	PDS	String
	Home circumstances	Onboarding	Categorical
	Smoker	Onboarding / GP	Categorical
	**Clinical Comorbidities:***		
	BMI	Onboarding / GP	Numeric
	Asthma	Onboarding / GP	Binary
	COPD	Onboarding / GP	Binary
	Emphysema	Onboarding / GP	Binary
	Bronchiectasis	Onboarding / GP	Binary
	Other Chronic Resp Disease	Onboarding / GP	Binary / string
	Chronic cardiac disease	Onboarding / GP	Binary
	Chronic liver disease	Onboarding / GP	Binary
	Stroke or dementia	Onboarding / GP	Binary
	Neurological	Onboarding / GP	Binary
	Chronic kidney disease	Onboarding / GP	Binary
	Hypertension	Onboarding / GP	Binary
	Diabetes	Onboarding / GP	Binary
	Cancer	Onboarding / GP	Binary
	Cancer site(s) / types	Onboarding / GP	String
	Date of Cancer Diagnosis	Onboarding / GP	Date
	HIV	Onboarding / GP	Binary
	Transplant receipient	Onboarding / GP	Binary
	Transplant sites	Onboarding / GP	Cateogrical
	Immunosuppression regimen	Onboarding / GP	String

	**Onset of Symptoms:**		

Initial Entry	Breathlessness	Onboarding	Date
	Myalgia	Onboarding	Date
	Chills	Onboarding	Date
	Severe fatigue	Onboarding	Date
	Sputum	Onboarding	Date
	Dizziness	Onboarding	Date
	Cough	Onboarding	Date
	Nausea or vomiting	Onboarding	Date
	Diarrhoea	Onboarding	Date
	Headache	Onboarding	Date
	Sore throat	Onboarding	Date
	Nasal congestion	Onboarding	Date
	Anosmia	Onboarding	Date
	Unwell contact	Onboarding	Binary
	Sars COV 2 swab positive	Onboarding	Categorical
	Referral source	Onboarding	Categorical
	Date and time of recording	Onboarding	Timestamp
	**Clinical observations:**		
	O2 sats at rest	Onboarding	Numerical
	O2 sats after exertion	Onboarding	Numerical
	Heart rate	Onboarding	Numerical
	Respiratory rate	Onboarding	Numerical
	Blood pressure	Onboarding	Numerical
	Conscious level (GCS)	Onboarding	Numerical
	Temperature	Onboarding	Numerical
	NEWS Score	Onboarding	Numerical

Diary Entry (iterates 2-3x daily)	Date and time of recording	Diary	Timestamp
	**Clinical observations:**		
	O2 sats at rest	Diary	Numerical

	O2 sats after exertion	Diary	Numerical
	HR	Diary	Numerical
	Temperature	Diary	Numerical

Diary Aggregate Data (N.B. Derived from Diary entries - not collected in itself)	*Mean pulse*	*Calculated from Diary*	*Numeric*
	Mean O2 sats	*Calculated from Diary*	*Numeric*
	Datetime of lowest O2 Sats	*Calculated from Diary*	*Numeric*
	Value of lowest O2 Sats	*Calculated from Diary*	*Numeric*
	Datetime of highest HR	*Calculated from Diary*	*Numeric*
	Value of highest HR	*Calculated from Diary*	*Numeric*
	Datetime of highest Temp	*Calculated from Diary*	*Numeric*
	Value of highest Temp	*Calculated from Diary*	*Numeric*
	Number of days of fever	*Calculated from Diary*	*Numeric*
	Days on Virtual Ward	*Calculated from Diary*	*Numeric*

In Hospital Data (one for each A&E presentation or hospital admission)	First location on arrival	Hospital EHR / HES	Categorical
	Date and time of arrival	Hospital EHR / HES	Timestamp
	Admitted from A&E	Hospital EHR / HES	Binary
	**Obs on arrival:**		
	O2 sats on air	Hospital EHR	Numeric
	O2 desturation (>3%)	Hospital EHR	Numeric
	Heart rate	Hospital EHR	Numeric
	Respiratory rate	Hospital EHR	Numeric
	Blood pressure	Hospital EHR	Numeric
	Conscious level (GCS)	Hospital EHR	Numeric
	Temperature	Hospital EHR	Numeric
	NEWS Score	Hospital EHR	Numeric
	**Treatment given:**		

	Highest Oxygen Requirement	Hospital EHR	Numeric
	High flow oxygen	Hospital EHR	Binary
	CPAP	Hospital EHR	Binary
	NIV	Hospital EHR	Binary
	Intubation	Hospital EHR	Binary
	level 2 care	Hospital EHR	Binary
	Level 3 care (ITU)	Hospital EHR	Binary
	**Outcomes:**		
	Date and time of discharge or in-hospital d	Hospital EHR / HES	Timestamp
	Discharge destination	Hospital EHR / HES	Categorical
	Length of Stay	*Computed from in-hospital data*	*Numeric*

Mortality	Date of Death	ONS	Date
	Cause(s) of Death	ONS	String

For peer review only

STROBE Statement—checklist of items that should be included in reports of observational studies

	Item No	Recommendation	Page No
**Title and abstract**	1	(*a*) Indicate the study's design with a commonly used term in the title or the abstract	2
		(*b*) Provide in the abstract an informative and balanced summary of what was done and what was found	2
**Introduction**			
Background/rationale	2	Explain the scientific background and rationale for the investigation being reported	3
Objectives	3	State specific objectives, including any prespecified hypotheses	3
**Methods**			
Study design	4	Present key elements of study design early in the paper	4
Setting	5	Describe the setting, locations, and relevant dates, including periods of recruitment, exposure, follow-up, and data collection	4
Participants	6	(*a*) *Cohort study*—Give the eligibility criteria, and the sources and methods of selection of participants. Describe methods of follow-up<br>*Case-control study*—Give the eligibility criteria, and the sources and methods of case ascertainment and control selection. Give the rationale for the choice of cases and controls<br>*Cross-sectional study*—Give the eligibility criteria, and the sources and methods of selection of participants	4
		(*b*) *Cohort study*—For matched studies, give matching criteria and number of exposed and unexposed<br>*Case-control study*—For matched studies, give matching criteria and the number of controls per case	N/A
Variables	7	Clearly define all outcomes, exposures, predictors, potential confounders, and effect modifiers. Give diagnostic criteria, if applicable	4
Data sources/ measurement	8*	For each variable of interest, give sources of data and details of methods of assessment (measurement). Describe comparability of assessment methods if there is more than one group	*4*
Bias	9	Describe any efforts to address potential sources of bias	4,5
Study size	10	Explain how the study size was arrived at	4
Quantitative variables	11	Explain how quantitative variables were handled in the analyses. If applicable, describe which groupings were chosen and why	4,5
Statistical methods	12	(*a*) Describe all statistical methods, including those used to control for confounding	5
		(*b*) Describe any methods used to examine subgroups and interactions	5
		(*c*) Explain how missing data were addressed	5
		(*d*) *Cohort study*—If applicable, explain how loss to follow-up was addressed<br>*Case-control study*—If applicable, explain how matching of cases and controls was addressed<br>*Cross-sectional study*—If applicable, describe analytical methods taking account of sampling strategy	N/A
		(*e*) Describe any sensitivity analyses	N/A

Continued on next page

For peer review only - http://bmjopen.bmj.com/site/about/guidelines.xhtml

**Results**			
Participants	13*	(a) Report numbers of individuals at each stage of study—eg numbers potentially eligible, examined for eligibility, confirmed eligible, included in the study, completing follow-up, and analysed	5
		(b) Give reasons for non-participation at each stage	5
		(c) Consider use of a flow diagram	N/A
Descriptive data	14*	(a) Give characteristics of study participants (eg demographic, clinical, social) and information on exposures and potential confounders	5,14
		(b) Indicate number of participants with missing data for each variable of interest	5
		(c) *Cohort study*—Summarise follow-up time (eg, average and total amount)	N/A
Outcome data	15*	*Cohort study*—Report numbers of outcome events or summary measures over time	
		Case-control study—Report numbers in each exposure category, or summary measures of exposure	
		Cross-sectional study—Report numbers of outcome events or summary measures	*6*
Main results	16	(*a*) Give unadjusted estimates and, if applicable, confounder-adjusted estimates and their precision (eg, 95% confidence interval). Make clear which confounders were adjusted for and why they were included	^
		(*b*) Report category boundaries when continuous variables were categorized	N/A
		(*c*) If relevant, consider translating estimates of relative risk into absolute risk for a meaningful time period	N/A
Other analyses	17	Report other analyses done—eg analyses of subgroups and interactions, and sensitivity analyses	5,6
**Discussion**			
Key results	18	Summarise key results with reference to study objectives	7
Limitations	19	Discuss limitations of the study, taking into account sources of potential bias or imprecision. Discuss both direction and magnitude of any potential bias	8
Interpretation	20	Give a cautious overall interpretation of results considering objectives, limitations, multiplicity of analyses, results from similar studies, and other relevant evidence	7-9
Generalisability	21	Discuss the generalisability (external validity) of the study results	8-9
**Other information**			
Funding	22	Give the source of funding and the role of the funders for the present study and, if applicable, for the original study on which the present article is based	10

*Give information separately for cases and controls in case-control studies and, if applicable, for exposed and unexposed groups in cohort and cross-sectional studies.

Note: An Explanation and Elaboration article discusses each checklist item and gives methodological background and published examples of transparent reporting. The STROBE checklist is best used in conjunction with this article (freely available on the Web sites of PLoS Medicine at http://www.plosmedicine.org/, Annals of Internal Medicine at http://www.annals.org/, and Epidemiology at http://www.epidem.com/). Information on the STROBE Initiative is available at www.strobe-statement.org.

For peer review only - http://bmjopen.bmj.com/site/about/guidelines.xhtml
